# Multiscale impact of researcher mobility

**DOI:** 10.1098/rsif.2018.0580

**Published:** 2018-09-26

**Authors:** Alexander M. Petersen

**Affiliations:** Ernest and Julio Gallo Management Program, Management of Complex Systems Department, School of Engineering, University of California, Merced, CA 95343, USA

**Keywords:** human mobility, propensity score matching, network science, science of science, researcher careers, diversity

## Abstract

International mobility facilitates the exchange of scientific, institutional and cultural knowledge. Yet whether globalization and advances in virtual communication technologies have altered the impact of researcher mobility is a relevant and open question that we address by analysing a broad international set of 26 170 physicists from 1980 to 2009, focusing on the 10-year period centred around each mobility event to assess the impact of mobility on research outcomes. We account for secular globalization trends by splitting the analysis into three periods, measuring for each period the effect of mobility on researchers' citation impact, research topic diversity, collaboration networks and geographical coordination. In order to identify causal effects we leverage statistical matching methods that pair mobile researchers with non-mobile researchers that are similar in research profile attributes prior the mobility event. We find that mobile researchers gain up to a 17% increase in citations relative to their non-mobile counterparts, which can be explained by the simultaneous increase in their diversity of co-authors, topics and geographical coordination in the period immediately following migration. Nevertheless, we also observe that researcher's completely curtail prior collaborations with their source country in 11% of the cross-border mobility events. As such, these individual-level perturbations fuel multiscale churning in scientific networks, e.g. rewiring the connectivity of individuals and ideas and affecting international integration. Together these results provide additional clarity on the complex relationship between human capital mobility and the dynamics of social capital investment, with implications for immigration and national innovation system policy.

## 1. Introduction

The dispersion of knowledge across institutional and national borders is fundamental to scientific progress. Historically, knowledge exchange has been mediated via the physical mobility of people and printed publications, however, it is progressively mediated by a combination of physical and virtual networks. While researcher relocation remains a prominent conduit for knowledge transfer, researchers can now choose from a variety of virtual alternatives to explore new research environments and collaborations. And while the professional prospects and call of adventure associated with relocation may be alluring to some, there are nevertheless risky trade-offs associated with physical relocation that require careful assessment of local versus non-local socio-economic, family, work and funding opportunities [1–4]. This common dilemma factors into the cost of human and social capital investment in science, which is rather substantial and continues to grow with the globalization of the scientific endeavour [1,5].

To stay or leave? We provide guidance on this pivotal question by quantifying the impact of researcher mobility from four perspectives—scientific impact, research topic diversity, collaboration and international integration—together providing insights into the socially mediated network of networks connecting individuals and knowledge production [6]. As such, our results contribute to the literature on how knowledge flows [7,8] and how careers grow [9] following pivotal events—e.g. winning the Nobel Prize [10], initiating a dedicated partnership [11], obtaining tenure [12] and pursuing cross-disciplinary migration [13]—which in turn mediate shifts in collaboration [14–16] and research topic exploration [17–20].

In particular, we focus on the domain of physics, a broad research field characterized by relatively high levels of geographical mobility, which contributes to growing efforts to measure the impact of high-skilled migration in both industry and academia [1–5,7,8,14–16,21–33]. We employ a data-driven approach, starting with author disambiguation of 355 808 research articles published in American Physical Society (APS) journals over the period 1980–2009, resulting in a final dataset of 26 170 prominent researcher profiles that are broadly distributed both in terms of geography, productivity and scientific impact.

We leverage the size and breadth of this researcher dataset to address common reverse causality and selection bias problems that render identifying causal mobility effects rather challenging [34]. For example, many studies to date focus on select subsets of migrant researchers—e.g. elite scientists, winners of prestigious international fellowships or participants in select national exchange programmes—in which measured shifts in performance may endogenously depend on the selection of migrant researchers being analysed. Instead, following a data-driven approach we are able to estimate the mobility effect within a diverse population, accounting for variation in time period, geographical region, and social and cognitive aspects of research activity.

Our results demonstrate the impact of researcher mobility across scales—from individual careers, to collaboration network churning, to international integration. As motivation, the Nobel Prize offers a prime example of multiscale impact, highlighting how countries with attractive academic and industrial R&D environments that support objective and impartial immigration policy are positioned to benefit greatly from the immigration of even just a single elite scientist capable of prosecuting ground-breaking and transformative research.

**Figure 1.**
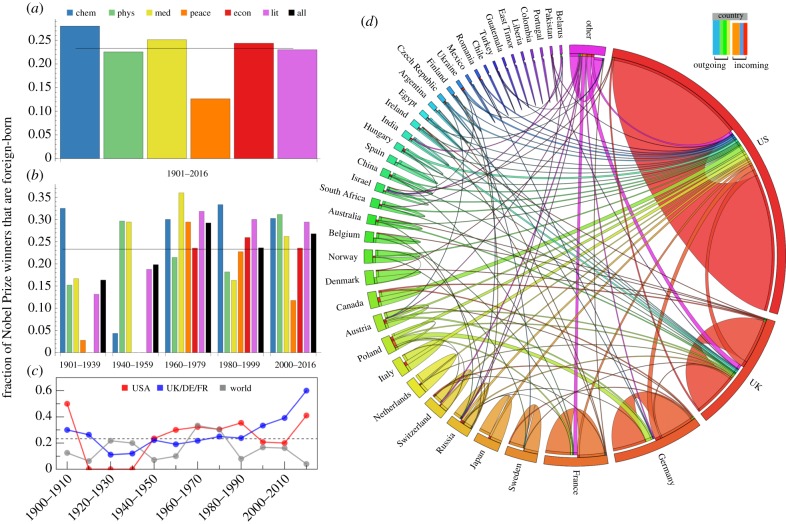
International mobility of Nobel laureates. (*a*) Fraction of Nobel Prize winners who received their award for work done in a country different than their birth country—‘foreign-born’; 23% of Nobel Prize winners from 1901 to 2016 belong to the foreign-born category, indicated by the horizontal line. (*b*) Fraction by award and time period. (*c*) Fraction by region of achievement over 10-year intervals. (*d*) The Nobelist birth-discovery network: countries are represented along the arc, with arc-length proportional to the number of laureates born in a given country. Link width represents the number of individuals born in country *c*_b_ that performed award research in country *c*_a_. The link direction is denoted by the gap, which differentiates incoming and outgoing links: e.g. the majority of the international links associated with the US terminate with a gap, indicating foreign-born. The majority of links are intra-country (e.g. 29% of all laureates have the US as their country of birth and their country of achievement), but the international links represent the nearly 1 in 4 Nobelists who migrated internationally. Data from *Wolfram Alpha* [35]. (Online version in colour.)

To provide insights into the global immigration patterns of elite scientists, we analyse the international pathways of all Nobel Prize winners through 2016 (figure 1). We found that roughly 1 in 4 are foreign-born—having performed their prize-winning research in a country different than their birth country. This finding is rather stable across the different award categories, with the exception of the Peace Prize, which has lower frequencies since there is a propensity for human rights activists to lead movements in their native countries. The UK, Germany and France, the three countries in Europe with the highest R&D spending, have seen a recent surge since the 1990s in the fraction of Nobelists from their country who are foreign-born. The rest of the world, not including the US, shows the opposite trend since the 1960s. There is also significant disparity in the flow counts from source to destination country, as figure 1*d* illustrates how research hubs like the USA, the UK and Germany attract a disproportionate share of foreign-born Nobelists.

While winning the Nobel Prize is likely to remain out of reach for most scientists, the bulk of scientists will nevertheless encounter an opportunity and be faced with the decision of whether to relocate. Based on our data, we estimate the rate of international mobility among all researchers in our dataset, independent of productivity level, to be roughly 2 in 5 (40%); and including US inter-state cross-border mobility this rate is closer to 3 in 5 (58%).

In what follows, we focus on several thousand relatively productive physicists across three decades, implementing statistical matching techniques that leverage the breadth of our dataset to compare researchers who migrate to similar researchers who do not. As such, we demonstrate the value of cross-border mobility on research outcomes, reflecting the pervasive and persistent value of physical mobility as a conduit for the exchange of intellectual, professional and cultural experience.

## 2. Results

### 2.1. Researcher data

We analysed the APS *Physical Review* datasets ‘Citing article pairs’ and ‘Article metadata’ [36], from which we extracted researcher profiles using a network-based author name disambiguation method [37]; see electronic supplementary material, appendix A.1 for additional details. Application of this algorithm identified 208 734 distinct researcher profiles over the 30-year period 1980–2009. Each researcher profile, indexed by *i*, is characterized by the year of researcher's first APS publication, *y*^0^_*i*_, and total number of APS publications *N*_*i*_. We concentrate on the 26 170 researchers with *N*_*i*_ ≥ 10 publications who also meet additional career longevity and productivity criteria. Electronic supplementary material, figure S1(A–C) provides a statistical summary of the publication and researcher profile data.

Despite the large sample size, there are several limitations common to large-scale data-driven approaches, which derive from missing contextual information at the researcher and geographical level. First, we lack data for physics and non-physics research published by these researchers outside of the set of APS journals. Yet because of the reputation and prominence of the APS journal family, we are able to make the assumption that researchers characteristic of physics-related domains are likely to publish in one or more of these journals rather frequently over the course of his/her career. Thus, as long as their publications in the APS journals are characteristic of their contemporaneous research output in other journals (albeit excluding the exceptionally novel and infrequent research published in high-impact multidisciplinary letters journals) then we do not expect this to significantly bias our estimates. In other words, our reported estimates measure the impact of mobility on typical research outcomes.

Second, and relevant to this previous point, in order to facilitate the precision of the statistical matching method used here that leverages the large size of our researcher sample, we only include relatively prolific APS researchers. This is achieved by thresholding on publication rates during the 10-year window around each mobility event, which also ensures that the publication measures are aggregated from sufficient sample size to be a reasonably robust measure of researcher attributes in the immediate (5-year) period before and after the event. However, this also means that we likely exclude many early stage (doctoral) and postdoctoral researchers who commonly migrate for brief periods, but do not have significant publication output to enter our study before they exit the academic career path. For example, according to US National Science Foundation Statistics, by 2008 the rate of US PhD recipients (postdoctoral fellows) in science and engineering who were foreign-born had grown to roughly 1 in 2 (3 in 5) [5]. Thus, our results do not capture the mobility premium for this highly mobile portion of the academic workforce, which deserves additional attention given shifts in the early and mid-career labour market [12,38–41] and the uncertainty associated with academic career trajectories [24,29,42–44].

And third, in addition to missing bibliometric information, we also lack other important information such as gender, which is an important factor relevant to career longevity and productivity in science [42,45–48], and possibly also migration decisions. In a broader sense, we lack contextual information on what *push and pull* factors underly researchers' decisions to migrate. For example, a 2011 survey analysis based on more than 15 000 researchers' responses reports that destination countries with competitiveness-oriented national science policy (i.e. oriented around ‘prestige’ and ‘research excellence’) is a major attractive factor [31]. A different study based on more than 10 000 biomedical researchers, which incorporates social and familial factors in additional to the traditional professional factors, also shows that researchers are attracted by the prospects associated with a competitive professional and peer environment; however, more accomplished researchers are less likely to move if they recently obtained NIH funding or if their children are in high school [3]. Countries with relatively high R&D expenditure levels tend to have higher rates of elite physicist immigration [2]. And researcher mobility between two countries is positively correlated with international student mobility in the opposite direction, indicative of the formation of cross-generational brain circulation channels [49]. Complementing these findings is an analysis of high-skilled migration within Europe over the period 1997–2014, showing that countries with higher government expenditure on education also make significantly more attractive destination countries [4].

### 2.2. Researcher mobility framework

The aim of our study is to measure shifts in researcher profiles before versus after an cross-border mobility event occurring in year t^*^_i,*T*_. Electronic supplementary material, figure S2 illustrates our framework for measuring the reconfiguration of research attributes before and after such a mobility event; see electronic supplementary material, A.1 for further details on the calculation of t^*^_i,*T*_. In particular, our framework relies on the following subscript notation: we split into three time periods (indexed by *T*), drawing on numerous researcher profiles (indexed by *i*), which are then split into three mobility groups (indexed by *G*). Moreover, in order to assess individual researcher profile attributes, we also analysed the collaborators, keywords, and countries associated with each researcher profile, hereafter generically indexed by *j*.

For two main reasons, we split the analysis into three mobility observation periods denoted by *T*, each defined by non-overlapping lower and upper year limits, *t*^−^_*T*_ and *t*^+^_*T*_: *T*_1_ ≡ [1990−1997], *T*_2_ ≡ [1998−2003] and *T*_3_ ≡ [2004−2007]. First, this separation facilitates drawing period-specific conclusions; considered together, they facilitate identifying trends in the mobility effect over time. Second, this choice provides a compromise on the issue of how to treat multiple mobility events for a given researcher. In principle, a researcher could migrate multiple times over a short period (e.g. characteristic of a short postdoctoral or sabbatical period). However, we are primarily interested in the shifts in research activity associated with the first mobility event within a reasonable time frame. Thus, for a given analysis period *T*, we use just the first t^*^_i,*T*_ associated with a sequence of mobility events. By partitioning our analysis into multiple *T*, we then allow for subsequent mobility events by the same *i* to also contribute to our analysis. Electronic supplementary material, figure S1(D) provides a schematic of the separation of the analysis into three observation periods: *R* is the number of researcher profiles analysed per *T*: *R*_1_ = 4124, *R*_2_ = 9362 and *R*_3_ = 13 457. The variable window size accounts for the growth of the profession, and the latter period is chosen to occur after 2004 so to account for the increased international mobility following the EU enlargement [4,16].

Electronic supplementary material, figure S1(E) provides a schematic description of our procedure for classifying each researcher *i* that was active in a given period *T* according to three groups: (i) *G*_*i*,*T*_ = 1 identifies researchers who did not migrate before or during period *T*; *G*_*i*,*T*_ = 2 identifies a ‘placebo’ mobility group comprised of researchers who were mobile prior to *T* but not during *T*; and (iii) *G*_*i*,*T*_ = 3 identifies researchers who were mobile during *T*, with specific mobility year t^*^_i,*T*_. Researchers in a given *T* belonging to either group *G*_*i*,*T*_ = 1 or 2 are prescribed t^*^_i,*T*_ ≡ Median[*t*^−^_*T*_, *t*^+^_*T*_], the midpoint of *T* (e.g. t^*^_i,1_≡1994). We then aggregated each researcher's publications in the Δ*t* ≡ 5-year window before t^*^_i,*T*_, i.e. over the interval [t^*^_i,*T*_ − Δ*t*, t^*^_i,*T*_ − 1]); likewise, we aggregated the publications after t^*^_i,*T*_ over the interval [t^*^_i,*T*_, t^*^_i,*T*_ + Δ*t* − 1]). This framework facilitates measuring research patterns over a balanced observation period for each *i*, thereby leveraging the longitudinal dimension of the researcher profiles. As a final dataset refinement, we excluded researcher profiles with fewer than three publications in either the period before or after t^*^_i,*T*_ and fewer than four distinct years of publication activity in total.

Electronic supplementary material, figure S1(F) shows the annual distribution of the total 31 075 mobility events, including in this count the multiple movements by a single researcher within a single *T*. Refining to just the first mobility event per researcher per period, we observe 6498 profiles belonging to the *G*_*i*,*T*_ = 3 group (i.e. roughly 21% of the total mobility events). These select researcher profiles are rich in data and sufficient in number to implement a matching method approach to estimate the impact of researcher mobility on research outcomes by comparing groups 1 and 3; moreover, we perform a robustness check by comparing researchers in groups 1 and 2 and researchers in groups 2 and 3. Intuitively, we expect that the mobility effect for the groups 1 and 3 comparison should be larger than the other group comparison estimates if our specification is to be considered consistent.

### 2.3. Research activity measures

We provide four complementary perspectives on (i) scientific impact, (ii) collaboration, (iii) research topics and (iv) geographical coordination, defined as follows:

#### 2.3.1. Citation impact

We normalized the standard integer citation count *n*_*i*,*p*,*t*_ for each publication *p* published in year *t* to account for temporal bias. The result is a normalized *z*-score *z*_*i*,*p*_, which is normally distributed for all *t*. We then calculate the average citation impact value *Z*^+,−^_*i*_≡〈*z*^+,−^_*p*,*i*_〉 across the *N*^+,−^_*i*_ publications in each Δ*t* ≡ 5-year interval, i.e. before (−) or after (+) t^*^_i,*T*_. We also use a measure of total citation impact, *Σ*^+,−^_*i*_, useful for assessing the magnitude of the mobility effect in real terms by applying a ‘citation deflator’ that accounts for ‘citation inflation’ [50].

#### 2.3.2. Co-author diversity

For each Δ*t* ≡ 5-year interval we count the number of articles published by *i* with co-author *j*, given by *k*_*ij*_. We then calculate the Shannon entropy for the distribution of *k*_*ij*_ across the set of *K*_*i*_ co-authors, defined as *E*^+,−^_*K*,*i*_. Higher levels of variation correspond to larger entropy values, with *E* ≥ 0; the limiting case of no variation, *k*_*ij*_ = const. for all *j*, corresponds to *E* = 0.

#### 2.3.3. Research topic diversity

Similar to (§2.3.2), we aggregate the instances of *Physics and Astronomy Classification Scheme* (PACS) codes associated with each publication to calculate a Shannon entropy measuring the variation in research topics, *E*^+,−^_*Q*,*i*_.

#### 2.3.4. Geographic reach diversity

We aggregate the countries indicated in the affiliation bylines and again calculate a Shannon entropy measuring the variation in geographical coordination, *E*^+,−^_*C*,*i*_.

See the Material and methods for further details on each measure. In what follows, we use the quantities *Z*^+^_*i*_, *Σ*^+^_*i*_, *E*^+^_*K*,*i*_, *E*^+^_*Q*,*i*_ and *E*^+^_*C*,*i*_ as dependent variables in five separate models, and the corresponding *Z*^−^_*i*_, *Σ*^−^_*i*_, *E*^−^_*K*,*i*_, *E*^−^_*Q*,*i*_ and *E*^−^_*C*,*i*_, along with five other covariate data measured before t^*^_i,*T*_, in order to match *i* between *G*_*i*,*T*_ groups. Electronic supplementary material, figures S3–S4 show the distribution of the quantities *Z*^+,−^_*i*_, *E*^+,−^_*K*,*i*_, *E*^+,−^_*Q*,*i*_ and *E*^+,−^_*C*,*i*_; see electronic supplementary material, A.2 and figure S5 for the analysis of the difference in these variables around t^*^_i,*T*_.

### 2.4. Reorganization of social and geographical links

When a researcher relocates, there is an immediate impact on his/her proximity to former collaborators. This reorganization of collaboration networks at the individual level can have far-reaching implications at the national and global scale. A case example is how East–West migration imbalance within Europe following the 2004 enlargement of the European Union (EU) [16] negatively impacted rates of international collaboration; it is not unlikely that high-skilled migration in response to Brexit will have a similar effect on international collaboration patterns in Europe [4].

Against this background, in this section we investigate the extent to which mobility mediates collaboration-based shifts in social and geographical integration, providing insights into the formation and disintegration of social capital. As above, *C*^−^ (*C*^+^) denotes the list of countries extracted from the publication affiliation byline (dropping the subscript *i* for brevity), with list length denoted by |*C*^−^| (|*C*^+^|). We also define the set of distinct countries as 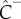 (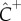), with lengths 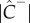 and 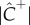, thereby disregarding the multiplicity of the countries in the original lists. By way of example, consider the hypothetical list of countries associated with the affiliations derived from an arbitrary set of publications, *C*^−^≡{CA, CA, FR, FR, AU, AU}; then the corresponding unweighted country list is 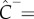
{CA, FR, AU}. These two definitions provide complementary perspectives on the degree to which geographical collaborations reorganize: the first measure (*C*^+,−^ ) is weighted proportional to the number of publications (i.e. per author affiliation), and the second is more aggregate (i.e. per country).

As such, we seek to quantify the degree to which the source country *c*^−^_*i*_ and destination country *c*^+^_*i*_ of a mobile researcher are related to *C*^+,−^. Thus, we define the ‘per-affiliation’ geographical association of the source (destination) country *c*^−^_*i*_ (*c*^+^_*i*_) with *C*^−^ (or *C*^+^) as2.1
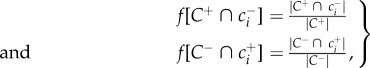
where |*X* ∩ *Y* | denotes the number of elements in the intersection of the two sets *X*, *Y*. Similarly, the ‘per-country’ measure of geographical association is defined using 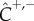 in equation (2.1) instead.

**Figure 2.**
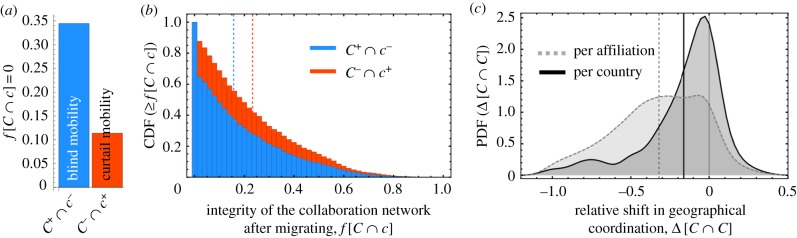
The impact of mobility on geographical collaboration networks. *C*^+^ ∩ *c*^−^_*i*_(*C*^−^ ∩ *c*^+^_*i*_) represents the geographical overlap between a researcher's location before (after) migrating, denoted by *c*^−^_*i*_(*c*^+^_*i*_), and the set of collaborator locations after (before) migrating, denoted by *C*^+^(*C*^−^). (*a*) Shown is the fraction of mobility events corresponding to: (i) ‘blind mobility’ (blue): in which there is no overlap between a researcher's prior location and the locations of future collaborators (*f*[*C*^+^ ∩ *c*^−^_*i*_ = 0] = 0.34); and (ii) ‘curtail mobility’ (red): in which there is no overlap between the locations of a researcher's prior collaborators and his/her destination country (*f*[*C*^−^ ∩ *c*^+^_*i*_ = 0] = 0.11). (*b*) The distributions of collaboration network integrity, measured by *f*[*C*^+^ ∩ *c*^−^_*i*_] and *f*[*C*^−^ ∩ *c*^+^_*i*_], are right-skewed: on average there is only a 16% overlap between *C*^+^ and *c*^−^_*i*_ and a 23% overlap between *C*^−^ and *c*^+^_*i*_. (*c*) The difference Δ[*C* ∩ *C*] measures the change in the amount of geographical overlap (see equation (2.2)), measured in two ways: per affiliation and per country. Negative values indicate more overlap before as compared to after. Both methods indicate relatively high levels of collaboration network disintegration following a mobility event: 89% (70%) of the values are negative when measuring per affiliation (per country). Shown are calculations on data aggregated across all three periods, *T*_123_; for analogous plots specific to a given period *T* see electronic supplementary material, figure S6. Vertical lines indicate distribution mean values. (Online version in colour.)

Figure 2*a* shows that roughly 34% of the mobility events were ‘blind’, corresponding to the value *f*[*C*^+^ ∩ *c*^−^_*i*_] = 0, i.e. the destination country was not in the sphere of prior collaborations. Similarly, roughly 11% of the migrations corresponded to the scenario in which the sphere of destination collaborations did not intersect with the source country (*f*[*C*^−^ ∩ *c*^+^_*i*_] = 0), corresponding to the maximal curtailing of prior collaborations.

In addition to these extreme cases, we find that most other mobility events are followed by significant collaboration reorganization. Figure 2*b* shows the cumulative distribution function (CDF) CDF(≥ *f*[*C*^−^ ∩ *c*^+^_*i*_]) and CDF(≥ *f*[*C*^+^ ∩ *c*^−^_*i*_]), with both distributions more concentrated around small values. In the case of *f*[*C*^−^ ∩ *c*^+^_*i*_]), this indicates a relatively small likelihood that researchers maintain prior collaborations when they move.

We also analysed the dynamics of geographical coordination, conditional on the source country, defined as the difference2.2
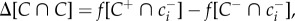
which is negative if there is more overlap between *c*^−^_*i*_ and *C*^−^ than between *c*^−^_*i*_ and *C*^+^. Figure 2*c* shows that most Δ[*C* ∩ *C*] are negative—i.e. migration is associated with significant churning within collaboration networks. Electronic supplementary material, figure S6 shows the same distributions for data disaggregated by *T*, demonstrating the stability of these observations over time.

### 2.5. Estimating the mobility effect using propensity score matching

To what degree does researcher mobility affect scientific impact, topical direction and collaboration? In this section, we describe the Rubin causal inference framework [51] as it applies to estimating the impact of cross-border mobility on various quantitative researcher career metrics. Using potential outcome notation, we estimate the *average treatment effect on the treated* (ATET),2.3
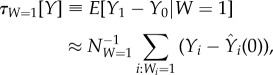
where *Y*_*i*_ indicates the outcome variable of interest, defined as one of the variables *Z*^+^_*i*_, *Σ*^+^_*i*_, *E*^+^_*K*,*i*_, *E*^+^_*Q*,*i*_, or *E*^+^_*C*,*i*_ and *E*[*Y*_1_ − *Y*_0_] denotes the expected difference between the two counterfactual outcome measures. The indicator *W*_*i*_ = 1 denotes ‘treatment’, i.e. mobility by individual *i* in period *T*; conversely, *W*_*i*_ = 0 corresponds to no mobility during *T*. Of course, a researcher is observed with either *W*_*i*_ = 0 or *W*_*i*_ = 1 in a given *T*, but not both; thus, the challenge is to estimate the counterfactual outcome, i.e. what would have happened had the researcher *i* not migrated. Hence, we use the propensity score matching (PSM) method [52] to identify researcher pairs (*i*, *i*′), where *i*′ is as similar as possible to *i* in terms of likelihood to belong to the treated group based on measured researcher characteristics (denoted by 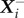) prior to t^*^_i,*T*_. Equation (2.3) defines the mobility-effect estimate *τ*_*W*=1_[*Y*] according to the PSM method, which approximates the counterfactual outcome 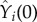 for researcher *i* with *Y*_*i*′_(0) by identifying the closest match *i*′ from the pool of *N*_*W*=0_ researchers with *W* = 0. In this way, *τ*_*W*=1_ is approximated as the average researcher-level effect across the sample of *N*_*W*=1_ researchers with *W* = 1. For a practical review of the matching estimators and their implementation see [53].

We estimated the treatment effect associated with each dependent variable *Y*^+^_*i*_ using a vector 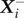 comprised of six covariates. We specified the logit model to calculate the likelihood of treatment for each *i*, which is used by PSM to match *i* with the closest *i*′ (results are reported for single matches, which we found to be consistent with nearest-neighbour matching up to the five closest matches). To be specific, for a given dependent variable *Y*^+^_*i*_, the six covariates we used to match are: (i) the outcome variable calculated for the period before the mobility, *Y*^−^_*i*_; (ii) the number of distinct co-authors, |*k*^−^_*ij*_|; (iii) the number of publications, *N*^−^_*i*_; (iv) the average citation impact, *Z*^−^_*i*_; (v) the researcher age s^*^_i_ = t^*^_i,*T*_ − *y*^0^_*i*_ + 1 in the year of the mobility event; and (vi) a factor variable *F*^−^_*i*_ which maps the country of residence *c*^−^_*i*_ to one of five geographical regions (N. America, S. & C. America, Europe, Asia & Australasia, Africa; see electronic supplementary material, A.3). Electronic supplementary material, figure S7 shows the distribution of each model variable and the corresponding correlation matrix.

Electronic supplementary material, table S1 shows the estimates of a logit model for the dependent variable 1_*G*_*i*_ = 3_, with value 1 if a researcher *i* belongs to the mobile group *G*_*i*_ = 3 and 0 otherwise, thereby estimating the mobility likelihood depending on a given researcher's attributes. See electronic supplementary material, appendix A.4 for analysis of the factors that correlate with cross-border mobility, along with refs. [1,3] which describe additional employment and other factors not feasible to include within our data-driven approach.

### 2.6. Impact of mobility on research outcomes

We applied the PSM method to five different dependent variables (*Z*^+^, *Σ*^+^, *E*^+^_*K*_, *E*^+^_*Q*_, *E*^+^_*C*_) calculated for three non-overlapping analysis time periods and for three different control-treated subgroup comparisons. More specifically, the matching between the groups G3 (treated, comprised of researchers with *G*_*i*,*T*_ = 3) and G1 (control, comprised of researchers with *G*_*i*,*T*_ = 1) provides the core estimate of the mobility effect; the matching between G2 (placebo-treated) and G1 (placebo control) provides a robustness check, as we do not expect there to be significant differences between the researchers in these two groups; and the matching between G3 (treated) and G2 (control), serves as an additional robustness check, since we hypothesize the mobility effect to be present, but to a lesser degree than the G3–G1 estimation.

**Figure 3.**
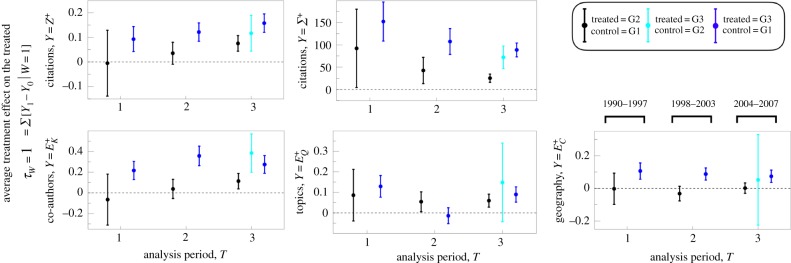
Estimation of the effect of mobility on various career measures. For a given variable *Y* and period of analysis *T*, we applied the PSM method to compare a ‘treated’ group (either *G*_*i*_ = 2 or 3) to a ‘control’ group (either *G*_*i*_ = 1 or 2), resulting in three comparison: G1–G2, G2–G3 and G1–G3. Each panel reports the results for a different dependent variable *Y*; shown are point estimates with error bar indicating 95% CI. (Online version in colour.)

Figure 3 reports the resulting 35 *τ*_*W*=1_[*Y*] mobility-effect estimations; insufficient samples sizes for the G2 and G3 groups limited the PSM matching performance for the periods *T*_1_ and *T*_2_ (see electronic supplementary material, figure S2(D) for the size of G1, G2, G3 for each *T*). To demonstrate model robustness, electronic supplementary material, figure S8 shows the estimates using the nearest-neighbour method (*nnmatch* in STATA13) instead of the PSM method (*psmatch* in STATA13); the important difference is that with the *nnmatch* method one can force an exact match on the source region *F*^−^_*i*_. The results using *nnmatch* are consistent with the results of PSM, with the exception of the ATET for *E*^+^_*K*_, which are significantly smaller in significance and magnitude. The Material and methods section summarizes additional robustness checks to further assess the statistical significance of our PSM estimates.

The PSM results indicate that researcher mobility has a significant positive effect on citation impact and increases diversity in research topics and collaboration at the individual and geographical level. Notably, the effect size for mean citation impact, *τ*_*W*=1_[*Z*^+^], increased over each period *T*. Because *z*_*p*_ ≡ (ln[*n*_*p*_ + 1] − *μ*_*t*_)/*σ*_*t*_ is a logarithmic transform of the citation count variable *n*_*p*_ (see Material and methods), the mobility effect for the average publication, in terms of the per cent increase in *n*_*p*_, is approximately 100 × 〈*σ*〉 × *τ*_*W*=1_[*Z*^+^] = 9% to 17% depending on *T*.

We also observe a decreasing trend for *τ*_*W*=1_[*Σ*^+^], which at first appears inconsistent with the trend for *τ*_*W*=1_[*Z*^+^]. However, the *Σ*^+^ measure suffers from right-censoring bias (publications analysed for later *T* have not the same amount of time to accrue citations as for publications from previous *T*) and so the negative trend is confounded by this bias; the value of the citation premium is nevertheless statistically significant and relevant in magnitude: *τ*_*W*=1_[*Σ*^+^] ≈ 90–150 citations depending on *T*, which provides an estimate for the aggregate mobility effect tallying over all research produced in the 5-year period after mobility. This is a lower-bound estimate because the APS dataset does not include citations from articles published in other journals, nor does *Σ*^+^ account for the citation tally from researchers' publications in non-APS journals. Thus, it is likely that *τ*_*W*=1_[*Σ*^+^] would be significantly larger if non-APS publications and citations were included.

Among the entropy variables, the mobility effect is largest for *τ*_*W*=1_[*E*^+^_*K*_] (diversity of co-authors) and *τ*_*W*=1_[*E*^+^_*C*_] (diversity of geographical coordination). The effect of mobility on *E*^+^_*Q*_ (diversity of research topics) is statistically significant for periods *T*_1_ and *T*_3_, but not for *T*_2_. These results quantify the extent to which mobile researchers facilitate a valuable interface for intellectual exchange. A prime example was the cross-disciplinary interface formed by computing and biology researchers in the genomics revolution, in which this constructive configuration facilitated the cross-pollination of methods and institutional know-how that were crucial for the success of the Human Genome Project [13].

We further estimated the mobility effect using OLS regression on matched researcher pairs (*i*, *i*′), which facilitates incorporating covariates 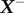 to explain the outcome variable *Y*^+^ associated with each *τ*_*W*=1_[*Y* ]. See electronic supplementary material, A.5 and tables S2–S6 for the description and results of this additional analysis. For example, we find that increasing researcher age correlates with lower research topic diversity, *E*^+^_*Q*_, suggesting that the ability to diversify into new research topics decreases with age (*β*_s*_ < 0; electronic supplementary material, table S5). We also observe a negative relation (*β*_s*_ < 0) between researcher age and citation impact variables, e.g. *Z*^+^ and *Σ*^+^, consistent with findings from previous studies on researcher careers [11,13].

**Figure 4.**
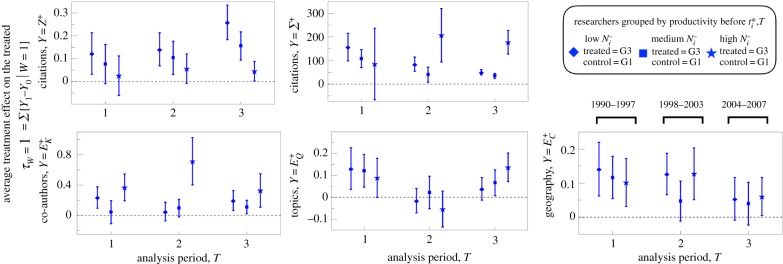
Estimation of the mobility effect by productivity subgroup. Researchers were separated into three groups according to the number of publications in the pre-treatment period, *N*^−^_*i*_: low (bottom tercile, triangles), medium (middle tercile, squares) and high (upper tercile, stars). For a given variable and period of analysis *T*, we applied the PSM method to compare the mobile ‘treated’ group (*G*_*i*_ = 3) to the not-mobile ‘control’ group (*G*_*i*_ = 1) corresponding to the group comparison G1–G3 in figure 3. Each error bar is a point estimate with 95% confidence interval. (Online version in colour.)

Are the observed mobility effects merely a feature of elite scientists in our dataset? To address this question, we separated the researchers into three terciles according to *N*^−^_*i*_ in order to further analyse the degree to which variation in *τ*_*W*=1_[*Y* ] is mediated by researcher productivity, a variable that is highly correlated with a researcher's characteristic team size, among other factors. Figure 4 reports the PSM estimates after matching researchers only within each subgroup, showing that the mobility effect on mean citation impact is largest for researchers belonging to the low- and medium-*N*^−^_*i*_ tercile groups; the mobility effect on co-author diversity is dominated by the high *N*^−^_*i*_ tercile group; and no significant variation is observed for topic diversity or geographical diversity.

## 3. Discussion

The globalization of science and decreasing costs of migration have democratized the opportunities for international mobility [2], thereby becoming an increasingly relevant topic for national innovation system policy [1], the economics of science [5], and the multiscale modelling of the scientific system [6]. Against this backdrop, we assembled and analysed a relatively comprehensive longitudinal dataset of researchers in physics in order to accurately quantify how researcher mobility affects career growth and intellectual exploration at the micro-level, and the churning of the collaboration network facilitating international integration at the macro-level. We used statistical matching techniques to measure the differences between mobile researchers and similar non-mobile researchers, thereby addressing the reverse causality and sample selection biases that hinder estimating causal effects. To this end, we developed a methodical framework for comparing the shifts in a researcher's publication profile in the 10-year period centred around each mobility event (see electronic supplementary material, figure S2 for a schematic of this framework).

We proceeded by separating our analysis into three time periods (*T*) in order to be able to identify significant temporal trends. Qualitatively, our quantitative results are not sensitive to *T*, indicating that the mobility premium identified for each variable (*Z*^+^, *Σ*^+^, *E*^+^_*K*_, *E*^+^_*Q*_, *E*^+^_*C*_) has not diminished as virtual avenues for exploring new collaborations proliferate. The results that are relevant to individual researchers are as follows. We measure a 9–17% increase in the number of citations received by articles published by mobile researchers, relative to the matched non-mobile control set. Aggregating this differential across all publications in the 5 years after migrating, on average this citation premium tallies up to the order of 100 citations (figure 3). A hypothetical mechanism that emerges directly from our analysis of the research diversity variables (*E*^+^_*K*_, *E*^+^_*Q*_, *E*^+^_*C*_) is that mobility increases the likelihood of drawing together different intellectual capacities and professional experience distributed across researchers, which when combined, provide a valuable perspective on how to best advance research efforts [17]. As such, this recombinant collaboration emerges as an important factor promoting recombinant innovation [54].

We complement this analysis based upon a broad cross-section of physicists with an alternative perspective based upon the extreme tail of scientific success—the 884 recipients of the Nobel Prize from 1901 to 2016. Analysis of their international trajectories reveals that 23% of these Nobelists were foreign-born, having received the award for work done in a country different than their birth country (figure 1). As such, these success stories provide ample evidence that just a single immigrant can have a monumental positive impact on a destination country, explaining to some degree the substantial international competition for elite scientists [2,3,5,21].

Motivated by recent work connecting migration imbalance (i.e. East–West ‘brain drain’) following the 2004 European Union enlargement to the stalled integration of national innovation systems across Europe [16,55], we also analysed the coevolution of mobility and collaboration from a systems perspective. In this regard, we provide additional micro-level evidence for such a mobility-mediated disintegration mechanism by showing that researchers completely curtail all former international collaborations roughly 11% of the time they migrate (figure 2). Conversely, we also found that 34% of the mobile researchers in our analysis moved to a location that was not in their sphere of prior collaborations. In addition to these exceptional cases, we observed remarkably high levels of churning following most mobility events. In this way, migration affords a prime opportunity to phase out old (possibly stagnant) collaborations and research topics in order to make way for exploring new (potentially lucrative) avenues. Other scholars have also noted that having migrated once likely increases the likelihood to migrate again, not merely due to additional experience and openness in regards to future opportunies, but also because recently shuffled collaboration networks are likely to be less restricting [31].

In conclusion, we analysed the dynamic interpersonal and international interface mediated by mobile researchers, one that facilitates the exchange of knowledge, as well as other valuable professional and cultural perspectives. While our focus was on mobile researchers in physics, our results are generalizable to other scientific and intellectual domains where cross-border experience confers a considerable comparative advantage by virtue of increased exposure to group diversity [56] and opportunities to broker knowledge [30], thereby promoting career development along an otherwise uncertain career path [24,29,42–44].

Against this background, recent scholarly efforts have provided considerable new insights into the professional, social and familial factors influencing scientists' decisions to stay or go [3,31,49]. For example, survey responses from more than 15 000 respondants indicated that ‘opportunities to improve future career prospects’ and ‘prestige/excellence of the institution’ were the two most important factors underlying their decision to move abroad [31]; however, a separate large study found that researchers were significantly less likely to relocate if they recently obtained competitive funding or if their children were in high school [3]. Related empirical work has also sought to determine whether other types of mobility—i.e. institutional, social, or inter-sectoral—produce a measurable effect on subsequent productivity and citation impact [28,57,58]; altogether these studies consistently report positive shifts in productivity, but a little if not insignificant effect on citation impact, when moving to a better institution.

International recruitment and home-return policy are typically geared around elite scientists [2,21,32], yet our results suggest that the career benefits of mobility are common to all ranks. Another important consideration is immigration policy, which can have a significant impact on the attraction and retention of talented researchers, thereby affecting the development of national innovation systems. An important example is the 2000 change in USA H-1B visa policy, which nullified the cap on available visas for non-profit sponsors, thereby eliminating the competition between industry and academia over this critical type of visa that facilities high-skilled immigration [5]. By analysing the annual changes in the number of H-1B visa recipients in industrial science and engineering, scholars found that higher rates of Chinese and Indian H-1B recipients correlated with higher levels of employment and patenting in innovation hubs that depend on immigrant high-skilled labour; moreover, the scholars report limited effects of the increase in H-1B immigrant population on native inventors' employment and patenting productivity [59]. These are important results showing that immigration of high-skilled labour does not appear to crowd out native employment opportunities and innovation capacity. And finally, countries with travel visa restrictions are negatively associated with international researcher mobility, in particular because they significantly increase the cost of travel, thereby inhibiting short-term collaboration visits that may precede the opportunity and ultimate decision to migrate long-term [49].

Considered in this way, our results contribute to these discussions by suggesting that national innovation systems and high-skilled immigration policy should not be so exclusively focused on the attraction and retention of elite scientists, but rather, should extend strategies to develop competitiveness by fostering international community and cultural diversity that serve all ranks of scientists. Moving forward on these issues, especially in consideration of the inherent difficulties—both ethical and statistical—in testing and empirically measuring the impacts of immigration policy [60], there is a need to develop better data-driven analytical and systems modelling methods to inform science policy on these issues. Indeed, anticipating the multiscale impacts of science policy is a formidable challenge calling for continued trans-disciplinary efforts to better understand the scientific enterprise [5,6,13,61].

## 4. Material and methods

### 4.1. Network-based author disambiguation method

We use the rich publication metadata in the APS dataset as input for a network-based author disambiguation method [37] that groups publications into researcher profiles. The Helbing algorithm leverages three key pieces of information available for each publication: (i) the co-author names, (ii) the publications listed in the reference list, i.e. outgoing citations, and (iii) the list of incoming citations from other publications (note that incoming and outgoing citations are restricted to publications within the APS dataset). Importantly, we did not provide any geographical affiliation information to the disambiguation algorithm, and so the resolved researcher profiles are free of geographical biases, and thus, well-suited for the study of cross-border mobility.

In an effort to assess statistically significant changes in publication profile attributes, and to reduce the frequency of spurious fluctuations arising from small sample size, we only analysed profiles with *N*_*i*_ ≥ 10 publications in the APS dataset and initial publication year *y*^0^_*i*_ ≥ 1985, resulting in 26 170 researcher profiles; electronic supplementary material, figure S1(C) shows the distribution *y*^0^_*i*_ for our final dataset, with 1995 as the median starting-year value.

### 4.2. Specification of research activity measures

#### 4.2.1. Citation impact

In order to compare the citation impact of publications (indexed by *p*) from different years (*t*), we apply a normalization method that maps the integer citation count *n*_*i*,*p*,*t*_ to a *z*-score using a logarithmic transform. To be specific, *z*_*i*,*p*_ ≡ (ln(*n*_*i*,*p*,*t*_ + 1) − *μ*_*t*_)/*σ*_*t*_, where *μ*_*t*_ = 〈ln(*n*_*t*_ + 1)〉 and *σ*_*t*_ = *σ*[ln(*n*_*t*_ + 1)] denote the mean and standard deviation calculated over the set of publications from a specific year cohort (*t*). By mapping citations to their log value (adding +1 to avoid the divergence associated with ln 0), and accounting for the age-cohort-specific mean and standard deviation, *z*_*i*,*p*_ follows a normal distribution *N*(0, 1) that is stable across *t* [11,13]. Publications with *z*_*i*,*p*_ > 0 are thus above the average log citation impact, *μ*_*t*_, and since they are measured in units of *σ*, standard intuition and statistics of *z*-scores apply. As such, *z*_*i*,*p*_ is well-suited for cross-temporal analysis, e.g. OLS regression, as well as averaging and summing within profiles. For this reason we calculate the average citation impact value 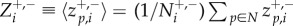 across the *N*^+,−^_*i*_ publications in each Δ*t* ≡ 5-year interval, before (−) or after (+) t^*^_i,*T*_. Moreover, due to the properties of logs, the per cent difference in *n*_*t*_ associated with the mobility effect is given by 100Δ*n*_*p*_/*n*_*p*_ = 100 × *σ*_*t*_ × (∂*z*/∂*W*) ≈ 100 × 〈*σ*〉 × *τ*_*W*=1_[*Z*^+^], which follows because *σ*_*t*_ is approximately constant over time, and so we approximate *σ*_*t*_ with the average value 〈*σ*〉 = 1.05.

For comparison, we also considered an ‘extensive’ citation impact measure, as opposed to *z*_*p*_, which is an ‘intensive’ impact measure. The deflated citation count 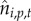 accounts for the fact that the total number of references produced by science is steadily growing with time (electronic supplementary material, figure S1(A)), the result of which is a ‘citation inflation’ measurement bias associated with the nominal citation count *n*_*i*,*p*,*t*_ [50]. However, the ‘deflated’ variable 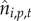 is well-suited for comparisons of citation counts for articles published in different years, and so we instead tally 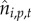 for each interval, defining the total as 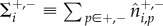. As such, *Σ*^+,−^_*i*_ is amenable to modelling differences in total citation impact before and after t^*^_i,*T*_.

#### 4.2.2. Co-authors

Within each Δ*t*≡5-year interval, *K*_*i*_ denotes the number of distinct co-authors we count for each *i*. Similarly, the number of publications including central researcher *i* and co-author *j* is *k*_*ij*_; the total number of co-author instances across all *N*_*i*_ publications is 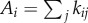. We then use the Shannon entropy, a diversity index measuring the variety across the *K*_*i*_ co-authors, defined as 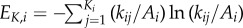. More specifically, we calculate the entropy *E*^−^_*K*,*i*_ (*E*^+^_*K*,*i*_) using data in the Δ*t*-year interval before (after) t^*^_i,*T*_. Higher levels of variation correspond to larger entropy values *E* ≥ 0; the limiting case of no variation, *k*_*ij*_ = const. for all *j*, corresponds to *E* = 0.

#### 4.2.3. Research topics

Similar to (§4.2.2), we aggregate the PACS codes associated with each publication, a system used by the American Institute of Physics and implemented broadly in physics journals since 1975; see https://publishing.aip.org/publishing/pacs/pacs-2010-regular-edition. This five-level classification is comprised of more than 5000 individual PACS codes, which authors self-assign to their publications (e.g. ‘89.75.-k’ corresponds to ‘Complex systems’); we observe on average 2.5 unique PACS per publication with only 1% of publications having five or more PACS. We aggregated the PACS codes from all publications in each observation period into the two lists denoted by *q*^+,−^_*j*_. We then define the variation *E*^+,−^_*Q*,*i*_ in each *q*^+,−^_*j*_ list of PACS codes using the same Shannon entropy measure in (ii) above.

#### 4.2.4. Geographical reach

As above, we aggregate the country codes associated with each publication affiliation into two lists, *C*^+,−^_*i*_. We define the variation *E*^+,−^_*C*,*i*_ in the categorical country code lists using the Shannon entropy (e.g. the list *C* = {NL, NL, NL, UK, IT, JP, AU} has entropy *E*_*C*_ = 1.475).

### 4.3. Robustness check of propensity score matching method

We randomized the treatment/control group classification variable 1_*G*_*i*_ = 3_ to test the likelihood that we could obtain an effect size as large as the observed *τ*_*W*=1_[*Y*] by chance. To be specific, we shuffled the treatment indicator 1_*G*_*i*_ = 3_ within each *G*3–*G*1 estimation, without replacement, thereby conserving the total number of observations (researchers) classified as being mobile during a specific period (i.e. belonging to group *G*_*i*_ = 3). We applied this shuffling procedure 10 000 different times, each time recording the value of the ‘placebo’ estimate for *τ*_*W*=1_[*Y*]. Figure S9 shows the distribution of placebo estimates, *P*(*τ*_*W*=1_[*Y* ]), for each *Y* ; in estimates, with one exception (panel D for *τ*_*W*=1_[*E*^+^_*Q*_]), we find the observed treatment effect to be significantly larger and outside the 99% CI bounds of the placebo estimate, thereby demonstrating the robustness of our PSM specification to spurious correlations.

## Supplementary Material

Supplementary Material

## Data accessibility

We used two principal open-source datasets provided by the American Physical Society [36] and WolframAlpha [35]; raw mobility data will be made available upon request.
