# Supplementary material for: Multiscale impact of researcher mobility
Source: J R Soc Interface. 2018 Sep 26;15(146):20180580. doi: 10.1098/rsif.2018.0580 (PMC6170771; doi:10.1098/rsif.2018.0580)
Supplement: Supplementary Material [file rsif20180580supp1.pdf]

Supplementary Material:

Multiscale impact of researcher mobility

Appendix, Figures S1-S9, and Tables S1-S6

Alexander M. Petersen[1]

[1] Ernest and Julio Gallo Management Program
Management of Complex Systems Department,
School of Engineering, University of California, Merced, CA 95343

S1. The American Physical Society dataset

We analyzed the 2009 American Physical Society (APS) *Physical Review article and citations* dataset, which is openly available in well-documented XML data files that record article-level author-byline data and PACS classification information for publications from the Physical Review journal family: *Physical Review A* (PRA), *Physical Review B* (PRB), *Physical Review C* (PRC), *Physical Review D* (PRD), *Physical Review E* (PRE), *Physical Review Letters* (PRL), and *Reviews of Modern Physics* (RMP). The publication metadata is homogenized and stable over time, and includes: (i) author name(s), (ii) affiliations with pointers to particular authors, (iii) citation data between APS articles, and (iv) Physics and Astronomy Classification Scheme (PACS) codes.

Data elements (i) and (iii) are inputs for the disambiguation of authors, detailed in the next subsection. It is important to note that the disambiguation algorithm we used does not use the affiliation metadata.. If the disambiguation algorithm *did* use the affiliation data, then there would be an increased likelihood of splitting researcher profiles according to intra-region publication clusters, which would not only increase the splitting (false negative) rate of researcher profiles into 2 or more clusters, but would under-represent the rate of researcher mobility. Instead, the clustering algorithm is not biased by geographic information contained in the affiliation data (ii). As a result, the publication clusters produced by the disambiguation algorithm – corresponding to disambiguated researcher profiles – are particularly amenable to geographic mobility analysis.

Author disambiguation method leveraging the collaboration and citation network. Because the PACS system was initiated in 1975, we include a 5-year buffer period before this year and the start year of our refined dataset. Hence, we analyzed 355,808 publications from 1980 – 2009. We then implemented the Helbing disambiguation algorithm [36]. This algorithm uses the citation network and the collaboration network to cluster publications into groups that are likely to correspond to an individual researcher. To be specific, the algorithm calculates a similarity score between any two given publications based on the overlap of (a) coauthor names, (b) the list of references cited by each publication, (c) the list of publications citing each publication, and (d) the particular scenario of direct citations between the two publications. This method was developed for large-scale data using the complete Web of Science dataset; Google Scholar profiles were used as a gold standard to obtain the algorithm parameters based on precision and recall error, in addition to several additional validation methods, including a theoretical model of the $h-$index distribution. Given the generality of this algorithm to scenarios in which the citation and collaboration network data are available, we applied it to the APS dataset using the optimal parameters reported in Shulz et al. [36].

More specifically, the algorithm works as follows. The starting point is the set of N_x publications that all list a given coauthor name, e.g. corresponding to the concatenated string A_x="LastName_FirstNameInitial" (e.g. Smith_A). We then calculate a similarity score between every pair of publications p and p' using a linear combination of weights for 4 factors: (i) the similarity

[1] Please send correspondence to: Alexander M. Petersen
E-mail: apetersen3@ucmerced.edu

in the set of coauthors, (ii) the self-citation scenario where on of p or p' cites the other, (iii) similarity in the reference lists of either publication, and (iv) similarity in the set of publications citing each publication. The algorithm first clusters the N_x publications into subgroups, and then merges subgroups into researcher profiles in a multi-step procedure.

Application of this algorithm produced A =208,734 publication clusters, indexed by $i = 1...A$, with each cluster containing N_i unique publications corresponding to the researcher profile of the author "LastName_FirstNameInitial$\#i$" (e.g. Smith_A#5). Figure S1(B) shows the distribution $P(N_i)$ of the number of publications per researcher profile.

Author selection procedure. We restricted our analysis to authors with greater than $N_i \geq 10$ publications spread over ≥ 3 distinct years and first publication $y_i^0 \geq 1985$; we implemented the last threshold to account for left censoring bias, i.e. to reduce the number of researchers in our analysis whose first publication was actually prior to 1980. As a final refinement, we excluded researcher profiles with fewer than three publications in either the period before or after $t^*_{i,T}$ and fewer than four distinct years of activity. The result of this additional selection is 26,170 APS researcher profiles corresponding to 206,272 distinct publications, which were cited 2,184,619 times altogether over their collective 986,287 years of citation activity. The total number of author career-year observations is 388,079, or roughly 15 career years per researcher profile.

Estimation of mobility year from raw publication data. The APS data has remarkably "clean" author affiliation data, which we used to geo-locate the individual articles by using string matches for country names, ISO2 and ISO3 country codes, and also the full names and 2-letter codes of US states which were used to classify affiliations that did not include "USA" but did include US State codes. Because the APS metadata has specific tags to link each researcher with one or more specific affiliations, we were able to link an individual i to specific countries and US states. When an author was affiliated with 2 or more countries in a given year, we tallied up these affiliations and assigned the primary location as the most common country within that year. In the case of a tie, we instead aggregated the affiliation data for the previous 3-year period and then used the most common country, which resolved 100% of the ties. Applying this geolocation method, we obtained an annual primary location time series for each author over the 30-year period 1980-2009; in the years in which the author did not publish in the APS dataset we denoted the primary affiliation as "blank". We then filled in the "blank" years in which the primary location before and after the blank period matched.

When the primary locations differ, before and after a period of $\delta y (\geq 0)$ "blank" years, this points to a mobility event. We estimate the mobility year t^*_i by first defining $y^+ \equiv y^- + \delta y$, where y^+ (y^-) is the first year after (before) the gap of "blank" years. If $\delta y = 1$ then we define the mobility transition year $t^*_i \equiv y^-$, and if $\delta y > 1$ then $t^*_i \equiv y^+ - \lceil \delta y/2 \rceil$.

S2. Research activity measures

Figures S3-S4 show the distribution of each model variable during the pre-mobility period $t \in [t^*_T - 5, t^*_T - 1]$ and post-mobility period $t \in [t^*_T, t^*_T + 4]$, respectively. We also calculated the change in the dependent variables for each researcher, between the pre- and post-treatment periods, as follows:

(i) *Citation impact*: We define the 2-period change in mean normalized citation impact as $\Delta Z_i \equiv Z^+_i - Z^-_i$.

(ii) *Coauthors*: As a measure of the 2-period change in the coauthor list, we calculate the similarity between the two lists using a variant of the cosine similarity, $S_{K,i} \equiv S[k^+_{ij}, k^-_{ij}] = (|k^+_{ij}||k^-_{ij}|)^{-1} \sum_j k^+_{ij} k^-_{ij}$, where $|k_{ij}| = \sqrt{(\sum_j k^2_{ij})}$ is the euclidian norm of the list in which the order of the categories (j) are matched so that they correspond to the same entity (e.g. coauthor) in k^+_{ij} and k^-_{ij}. Since $k_{ij} \geq 0$, $S[k^+_{ij}, k^-_{ij}] \in [0, 1]$, with maximum correspondence only when $k^+_{ij} = k^-_{ij}$ for all j.

(iii) *Research topics*: As in (ii) we measure the change in the PACS lists using the similarity distance $S_{PACS,i} \equiv S[q^+_j, q^-_j]$.

(iv) *Geographic reach*: As above we measure the change in the list of country codes drawn from the affiliation lists of each publication using the similarity distance $S_{C,i} \equiv S[C^+_j, C^-_j]$.

Figure S5 shows the distribution of ΔZ_i, $S_{K,i}$, $S_{PACS,i}$, and $S_{C,i}$, measuring the characteristic scale of research profile shifts, before and after $t^*_{i,T}$.

S3. Country classification

We classified countries into geographic regions as follows (2-letter ISO codes followed in parenthesis by the number of affiliations recorded for a corresponding country):

- **[Africa]**: ZA (1758), MA (204), EG (199), DZ (99), CM (61), TN (41), ET (17), NG (12), NA (5), LY (5), KE (4), MG (2), ZW (2), TZ (1), BI (1), GA (1), LS (1), GN (1), BW (1)
- **[Asia & Australasia]**: JP (155447), CN (38023), IN (30066), KR (26298), TW (18053), AU (14441), HK (4249), SG (2226), NZ (1661), AM (1256), IR (598), PK (325), UZ (318), PH (231), SA (224), VN (219), KZ (192), BD (143), TH (118), ID (78), MY (77), LB (53), JO (45), QA (44), KW (43), MN (43), AE (36), AZ (31), GE (29), OM (9), MO (5), BH (4), KG (3), IQ (3), PS (2), NP (1), SY (1)
- **[Europe]**: DE (151210), IT (147885), FR (109531), UK (98944), ES (31843), NL (25129), SE (18397), BE (10623), DK (9144), AT (8702), FI (8376), GR (4330), PT (2914), IE (1911), LU (18), PL (16132), HU (5424), CZ (4935), SI (3898), RO (2155), SK (1288), BG (1087), LV (275), LT (247), EE (238), CY (136), MT (5), CH (96970), RU (54597), IL (17797), NO (3935), UA (2939), HR (1913), YUGO (1260), TR (1240), CS (688), BY (418), RS (384), ME (109), IS (104), MD (84), MK (42), JE (8), AL (2)
- **[North America]**: USA (1,360,653), CA (63645), MX (6628)
- **[South America, Central America, and Carribean]**: BR (24304), AR (7553), CO (2022), CL (1573), VE (568), EC (235), CU (230), UY (187), PE (51), CR (14), BO (13), JM (12), PA (5), GD (4), BB (2), GY (1)

We classify the origin country (c_i^-) according to 5 broad regions, denoted by the factor variable F_i^- in the Propensity Score Matching and regression model specifications: (a) Europe; (b) N. America; (c) Central America, South America and the Caribbean; (d) Asia/Australia and (e) Africa. Because there were not many researchers from Africa with sufficiently large publication profile to meet our pruning criteria, observations associated with this region were excluded from our model estimates.

S4. Modeling mobility with the Logit model

We analyzed the factors that correlate with mobility in period T by modeling the dependent binary indicator variable $1_{G_i=3}$ – which takes the value 1 if $G_i = 3$ and 0 otherwise – by applying Logistic regression. This Logit model is specified within the Propensity Score Matching method to identify matched pairs [51]. We focus on just two sets of researchers for a given period, those researchers with $G_i = 1$ (not mobile up to and including the upper limit year t_T^+ of the period T) and $G_i = 3$ (mobile in T). Thus, we model the likelihood $P(G_{i=3})$ that a researcher is mobile given his/her research profile information, and so the binary outcomes follow the simple relation $P(G_i = 3) + P(G_i = 1) = 1$. For each i we included 5 variables measured, as previously, for the $\Delta t \equiv 5$-year period before $t^*_{i,T}$: the number of distinct coauthors, $|k_{ij}^-|$, the number of publications, N_i^-, the mean citation impact Z_i^-, the researcher age, s_i^*, and a factor variable representing the researcher's geographic region, F_i^-.

We model the odds $O \equiv P(G_i = 3)/P(G_i = 1)$ according to the Logit regression model specified as

$$\log\Big(\frac{P(G_i=3)}{P(G_i=1)}\Big) = \beta_1|k_{ij}^-| + \beta_2 N_i^- + \beta_3 Z_i^- + \beta_4 s_i^* + \beta_0 + F_i^- + \epsilon\ , \quad \text{(S1)}$$

which we estimate using robust standard errors. Table S1 reports the exponentiated coefficient, $\exp(\beta)$, which is the odds ratio, or factor by which the odds O changes for each 1-unit increase in the corresponding independent variable, i.e. $O_{+1}/O = \exp(\beta)$; put another way, $100(\exp(\beta)-1)$ is the percent change in O corresponding to a 1-unit increase in the corresponding independent variable. As a result, reported $\exp(\beta)$ values that are less than (greater than) unity indicate variables that negatively (positively) correlate with cross-border mobility.

The results of the model show that more coauthors correlate with a marginally smaller likelihood of migration for all T. Higher productivity (N_i^-) and citation impact (Z_i^-) correlate with a statistically significant higher likelihood of migration for T_1 and T_2 but not T_3, suggesting that mobility is becoming less contingent on researcher prestige. The most significant correlate is researcher age, which indicates a strong and statistically significant negative relation between increasing research age and likelihood of migration, observed for all T. The factor variables capturing the geographic region of residence prior to mobility (F_i^-) indicate that, relative to N. America (the most likely to migrate), a researcher residing in S. & C. America is the second most likely to migrate, followed by researchers from Europe, and then Asia & Australasia, in that order.

S5. Matched regression

While Propensity Score Matching is suitable for estimating the impact of treatment on post-treatment outcomes, it does not provide guidance as to the causal link between certain pre-treatment factors and the differential outcome. In order to estimate the degree to which certain researcher variables prior to $t^*_{i,T}$ correlate with the same set of variables after $t^*_{i,T}$, we used the set of matched researcher pairs (i, i') identified by the Propensity Score Matching method to regress each outcome (dependent) variable Y_i^+ against the set of pre-treatment matching variables denoted by $\vec{X}$. For example, in the first case where $Y_i^+ \equiv Z_i^+$, we regressed the post-migration average citation impact, Z_i^+, against the pre-migration variables $\vec{X} = (Z_i^-, |k_{ij}^-|, N_i^-, s_i^*, F_i^-, 1_{G_i=3})$, where F_i^- indicates a factor variable for the researcher's geographic sub-region (determined by the home country c_i^- prior to $t^*_{i,T}$), and $1_{G_i=3}$ is a binary indicator variable equal to 1 if the researcher migrated in period T and 0 otherwise. We performed OLS regression on the set of matched observations (i, i') according to the linear model

$$Z_i^+ = \beta_1 |k_{ij}^-| + \beta_2 N_i^- + \beta_3 Z_i^- + \beta_4 s_i^* + \beta_5 1_{G_i=3} + \beta_0 + F_i^- + \epsilon \,. \tag{S2}$$

Table S2 shows the results in columns (1,3,5) for each sample period T, respectively. The coefficient $\beta_5 \approx \tau_{W=1}[Y \equiv Z]$ reported in Fig. 3 for each T. That is, the treatment effect calculated by estimating the mean pairwise difference $Y_i - Y_{i'}$ between the matched researcher pairs (see Eq. [3]) is consistent with the difference in Z_i^+ between the two groups, controlling for $\vec{X}$. That is, the PSM treatment effect estimate is not confounded by $\vec{X}$.

Similarly, the model estimates reported in columns (2,4,6) of Table S2 correspond to the same model but including additional interaction terms between the scalar variables and the mobility indicator variable,

$$Z_i^+ = (\beta_1 |k_{ij}^-| + \beta_2 N_i^- + \beta_3 Z_i^- + \beta_4 s_i^*) \times 1_{G_i=3} + \beta_5 1_{G_i=3} + \beta_0 + F_i^- + \epsilon \,. \tag{S3}$$

As a result, this model specification yields two coefficients for each interacted covariate, one coefficient ($\beta_{x,G_i=3}$) derived from observations with $G_i = 3$ and a second coefficient ($\beta_{x,G_i=1}$) derived from those with $G_i = 1$. Tables S2-S6 report the coefficient $\beta_{x,G_i=1}$ followed by the difference in the two coefficients $\delta_3(x) \equiv \beta_{x,G_i=3} - \beta_{x,G_i=1}$, which facilitates identifying covariates that distinguish the mobile/treated (G3) and not-mobile/untreated (G1) groups.

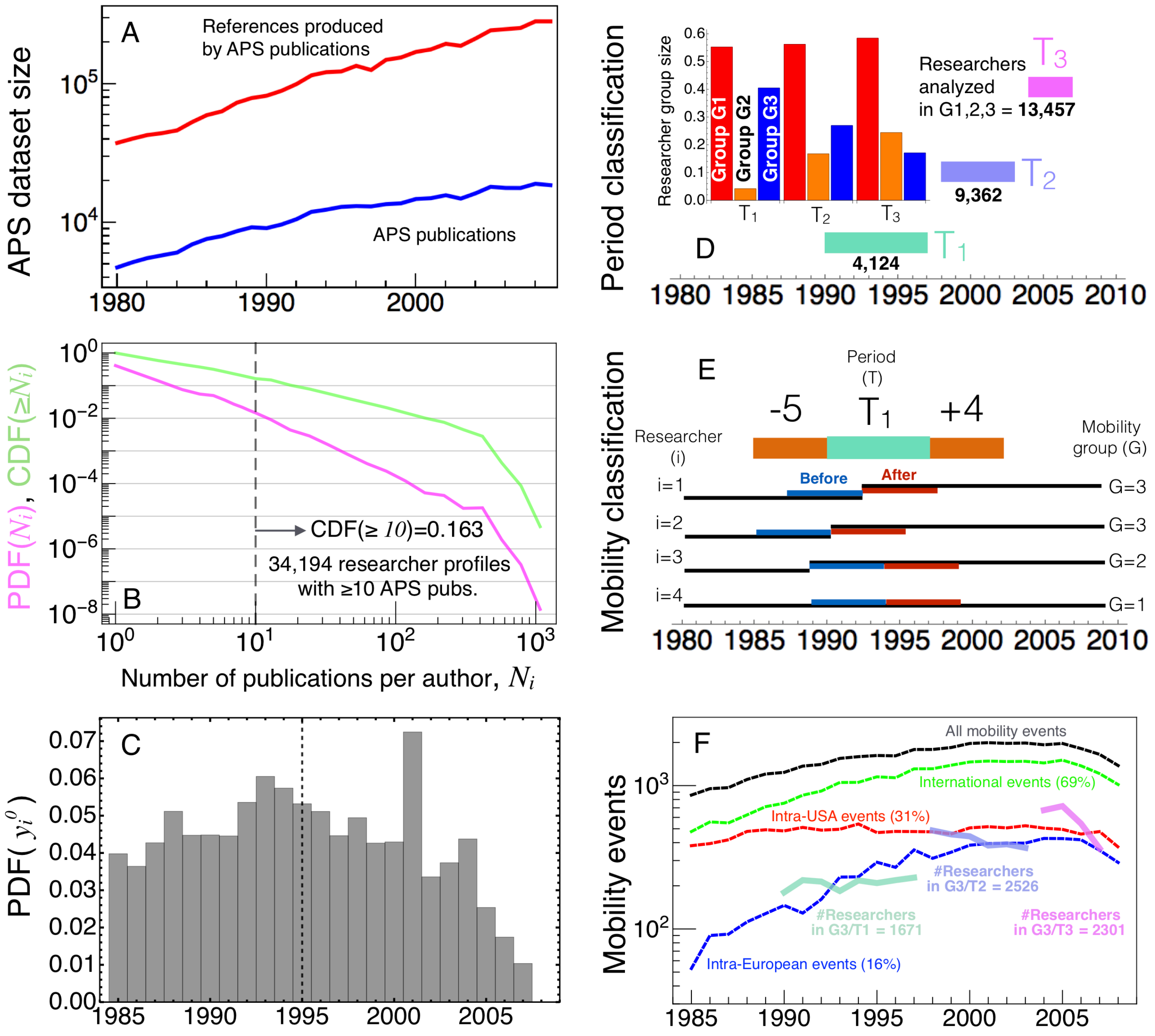

FIG. S1: **Data summary and 3-period observational framework**. **(A)** The total number of publications per year across the APS journals PRA, PRB, PRC, PRD, PRE, PRL, and RMP (blue), and the total number of references made by these publications that cite other APS publications within this journal set (red). Combined, the total number of publications is growing at roughly a 4.6% annual rate, and the total number of references made is growing at roughly a 7.2% annual rate over 1980–2009. **(B)** The distribution $P(N_i)$ of APS publications per researcher profile; 16.3% of the disambiguated researcher profiles have $N_i \geq 10$ corresponding to 34,194 profiles. **(C)** Distribution of researcher profiles according to their first APS publication year. We only analyzed researcher profiles with $y_i^0 \geq 1985$ and $N_i \geq 10$ publications spread across at least 3 distinct years, resulting in a total of 26,170 profiles. **(D)** We separated the mobility analysis into 3 non-overlapping observation periods, denoted by T, ensuring that each researcher contributes to the analysis of each T just once. (inset) Shown are the fraction of researchers belonging to a given mobility group G_T for a given T. The total number of researcher profiles by period are: 4,124 in T_1; 9,362 in T_2; 13,457 in T_3. Researchers (indexed by i) from the same T but different G are paired in the PSM analysis in order to estimate counterfactual outcomes. **(E)** Schematic of the classification process for 4 researcher profiles with respect to the observation period T_1: researchers 1 and 2 were mobile (indicated by the disjoint line) within the T_1 interval – thus they both belong to group $G3$, and so we aggregate the publication data in the 5-year window before and after the mobility event specific to each i; researcher 3 was mobile prior to T_1 but not during T_1, and so we use the midpoint of T_1 as a placebo mobility year and aggregate his/her publication data before and after the midpoint year of T_1 and assign this researcher to the placebo group $G2$; researcher 4 was neither mobile prior to nor during T_1, and thus belongs to the group $G1$. **(F)** Dashed lines correspond to the number of mobility events observed per year, allowing for multiple events per researcher profile: (blue) Intra-European32 mobility (e.g. DE to FR; EU32 corresponds to 28 EU members and CH, NO, LI, and IS); (red) Intra-USA state mobility (e.g. MA to CA); (green) International mobility (e.g. IT to USA); (black) All cross-border mobility, including both international and also inter-US state. Solid lines correspond to the number of mobile researchers in $G3$ by each period T. Note that even if a mobile researcher moved two or more times in a given T (i.e. multiple mobility events), this latter $G3$ researcher tally only counts these researchers once.

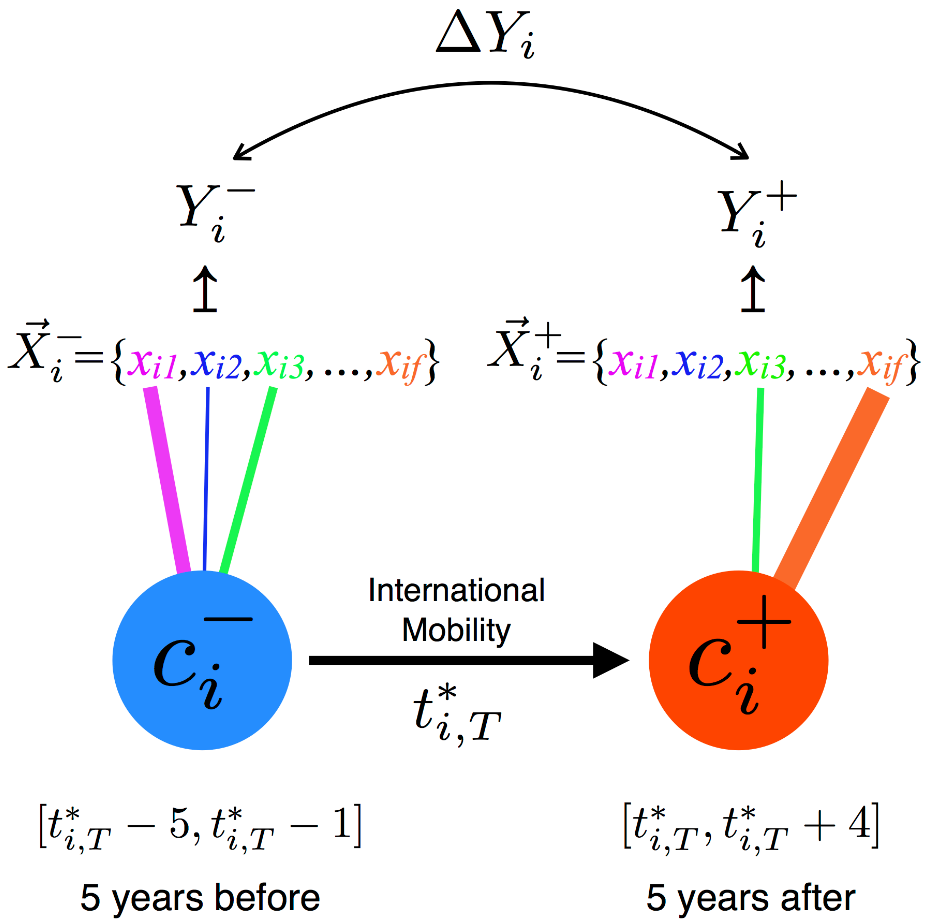

FIG. S2: **Schematic of researcher mobility framework**. For each researcher i we record their attributes $\vec{X}_i^{+,-}$ during the 5-year periods before and after the mobility event occurring in year $t^*_{i,T}$, from country c_i^- to country c_i^+. The weighted element x_{ij} represents a particular attribute, which by way of example, may be the number of publications with a particular collaborator, the number of instances of a particular PACS "keyword" capturing research topics, or other attributes of a single publication such as its citation count n_p or the set of countries C_p listed in the affiliation byline. We define a summary outcome variable $Y_i^{+,-}$, determined by particular information contained in $\vec{X}_i^{+,-}$, which facilitates: (a) measuring the change ΔY_i in the researcher profile attribute; (b) matching mobile and non-mobile researchers according to $\vec{X}_i^-$ and Y_i^- in order to obtain a causal estimate of the impact of mobility on Y_i^+.

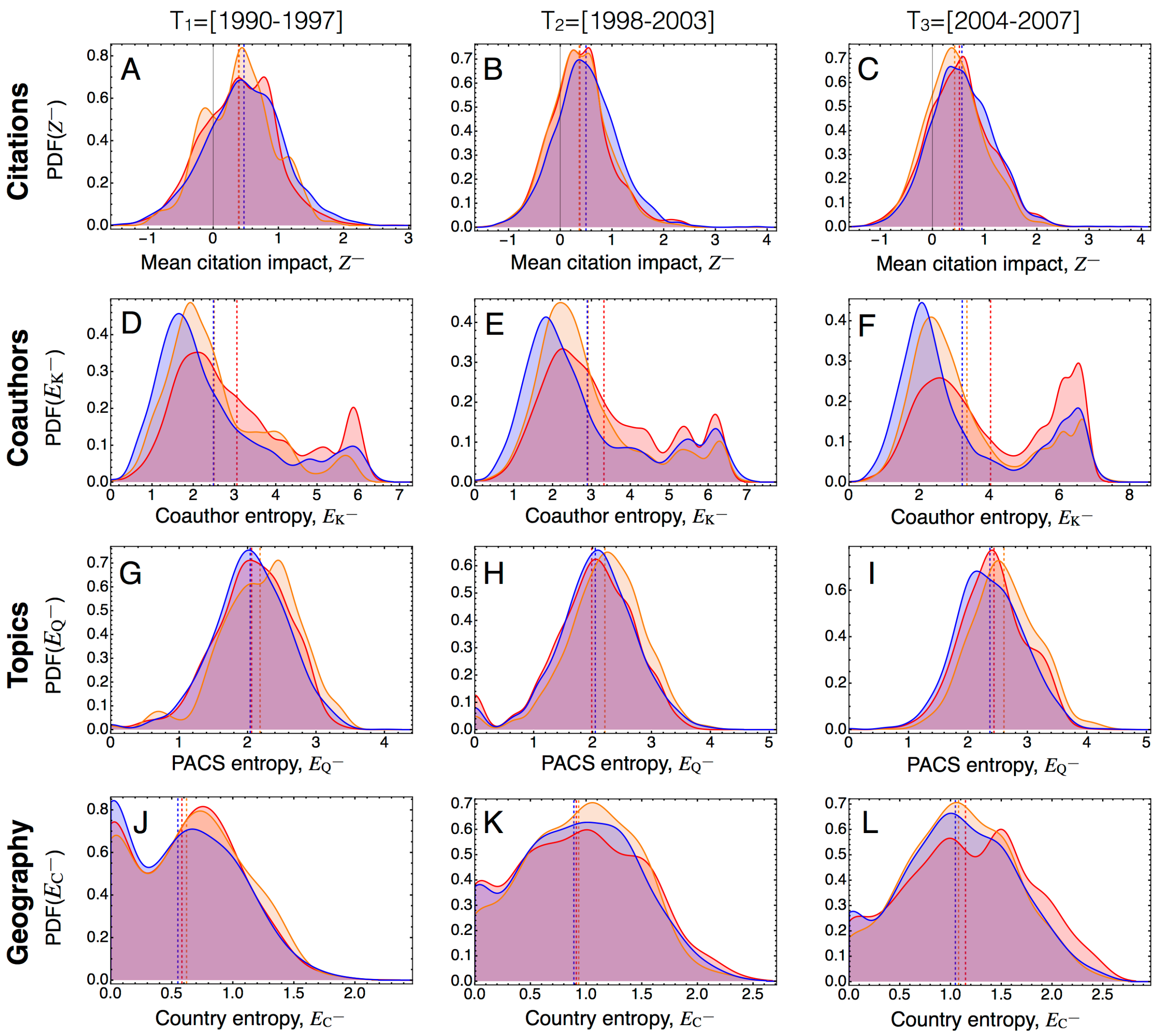

FIG. S3: **Distribution of PSM dependent variable values – before t^{*} by period**. Distributions demonstrate a high degree of stability between the three subgroups $G1$ (red: not mobile prior to the end of T), $G2$ (orange: mobile prior to the beginning of T but not mobile during T), and $G3$ (blue: mobile during T).

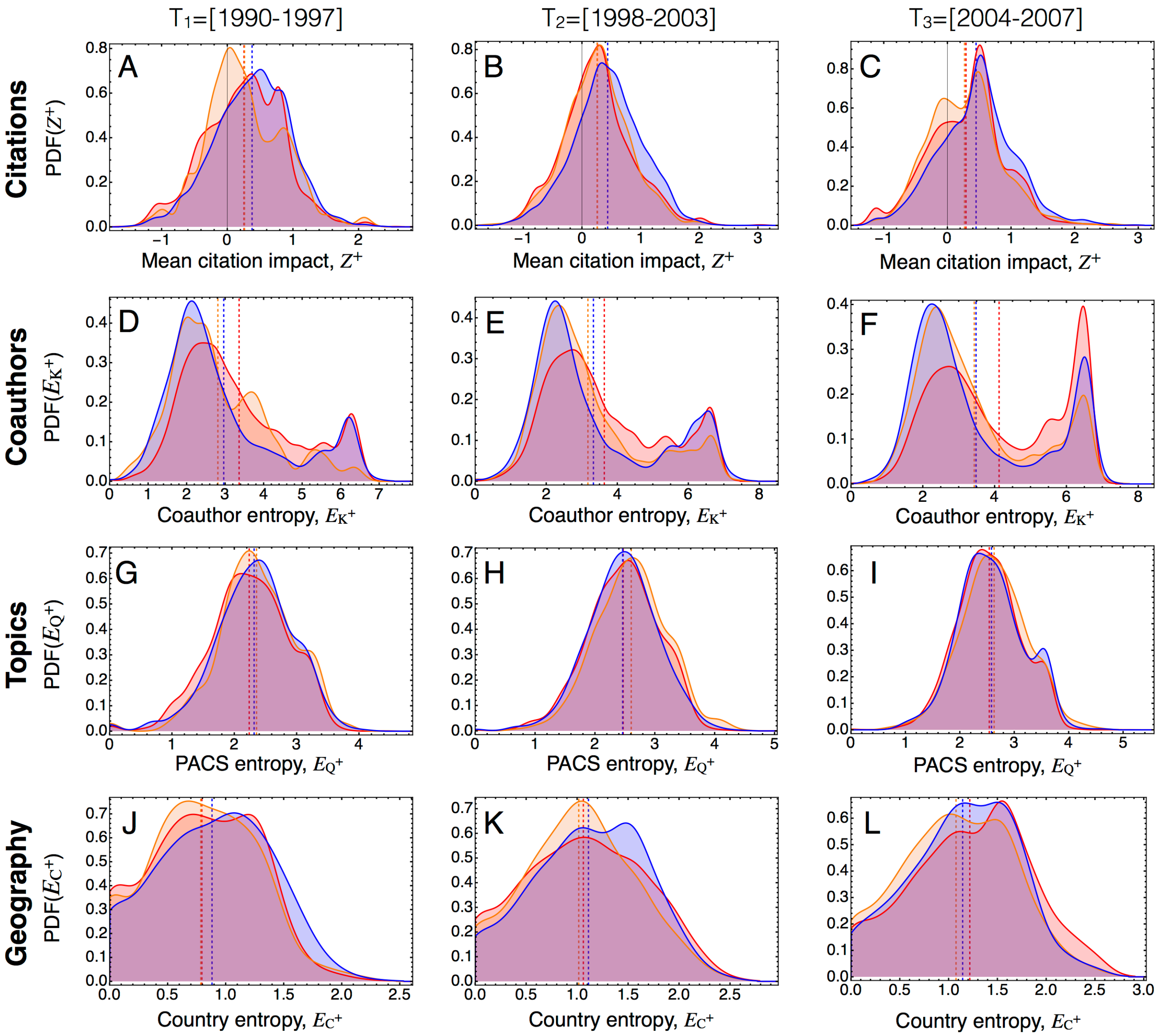

FIG. S4: **Distribution of PSM dependent variable values – after t^* by period**. Distributions demonstrate a high degree of stability between the three subgroups $G1$ (red: not mobile prior to the end of T), $G2$ (orange: mobile prior to the beginning of T but not mobile during T), and $G3$ (blue: mobile during T).

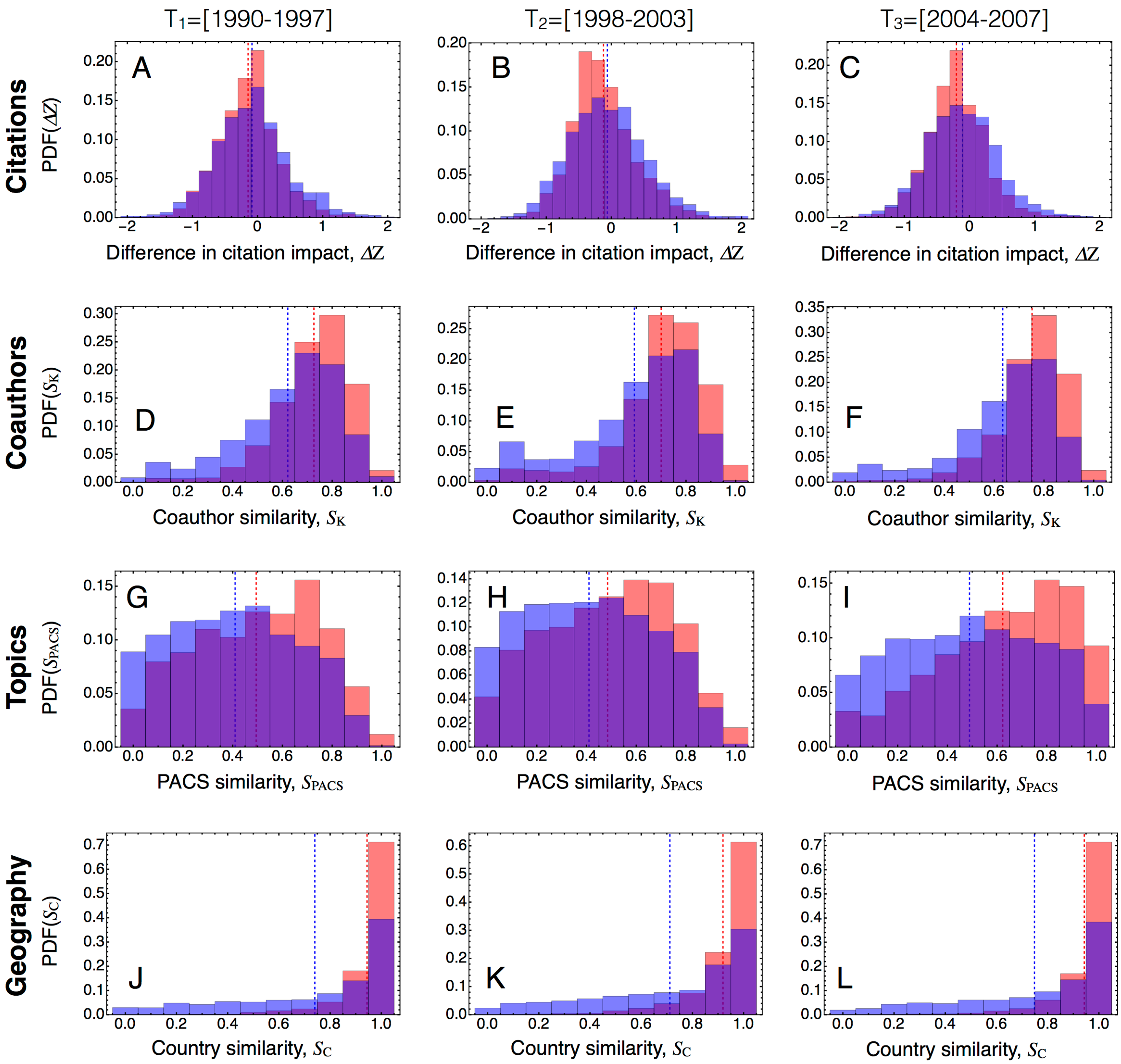

FIG. S5: **Distribution of change in career measures – before and after t^* – by period**. Each panel shows the probability distribution of a given quantity by mobility group and observation period. Comparison between groups $G1$ (no prior mobility, red) and $G3$ (mobility in period T, blue) provides an unconditional estimate of the impact of mobility on researcher trajectories in a given T. All variables measure the change in a given variable *after minus before* t^*_T. **(A-C)** Change in the citation impact: on average, researchers in the mobile group have slightly more positive change in citation impact. **(D-F)** Change in the collaborator network. **(G-I)** Change in the PACS research topics. **(J-L)** Change in the geographic network. For (D-L), one average, the mobile researchers have less similarity between their coauthors/topics/geography after migrating as compared to before migrating, than researchers from the control group.

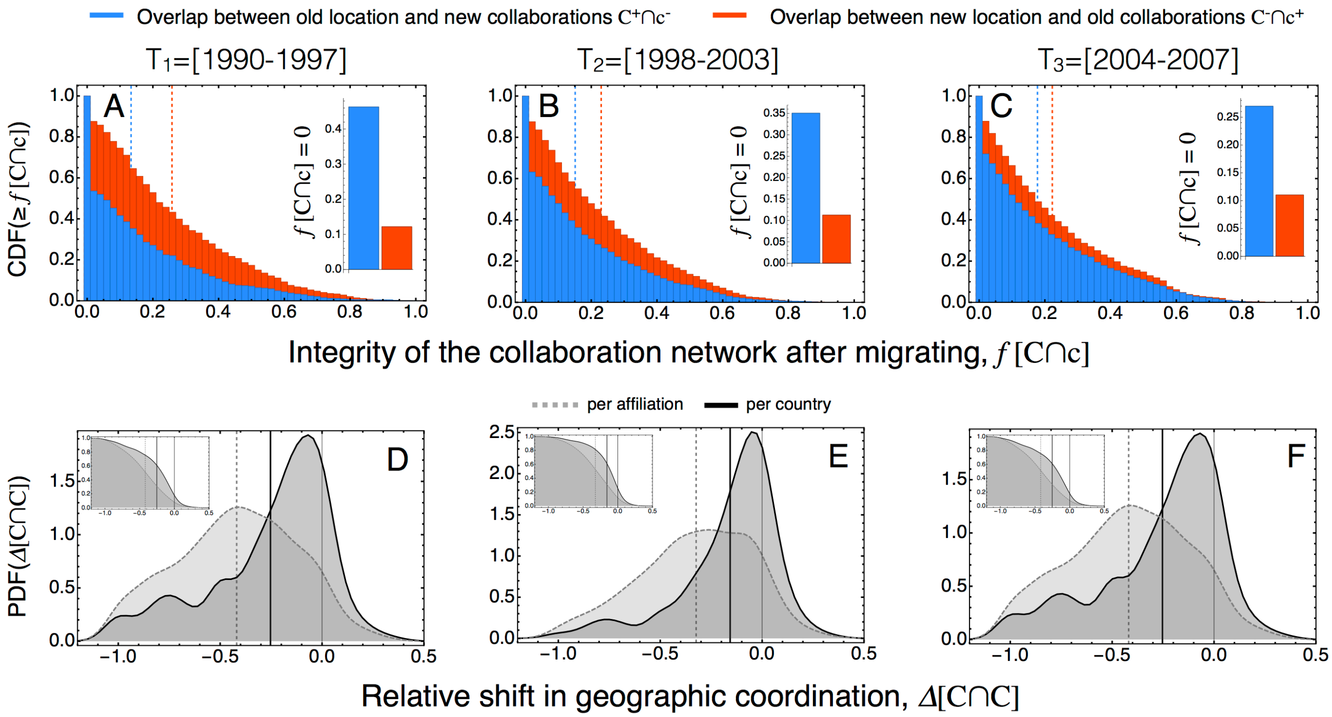

FIG. S6: **The impact of mobility on the geographic projection of collaboration networks – by period**. These results are calculated using data specific to each indicated period, thereby demonstrating the robustness of the distributions over time; compare with Fig. 2. **(A,B,C)** The degree of collobaration-mediated mobility measures the similarity between source and destination country of each i and the geographic distribution of his/her collaborators, before and after t^* – small values indicate the relatively low levels of similarity. (inset) Comparison of the "blind mobility" and "curtail mobility" rates. **(D,E,F)** Probability distribution of $\Delta[C \cap C]$ which measures the change in the geographic association between the collaborators before and after with respect to the source country of mobility, c_i^-. Negative values indicate that there is less overlap between c_i^- and the collaborators after the mobility event. For robustness, we calculate the geographic overlap in two ways: using distinct country lists (per country) and allowing for multiplicity due to multiple affiliations per publication (per affiliation). (inset) Cumulative probability distribution indicating that the majority of $\Delta[C \cap C]$ values are negative. Vertical lines indicate mean values.

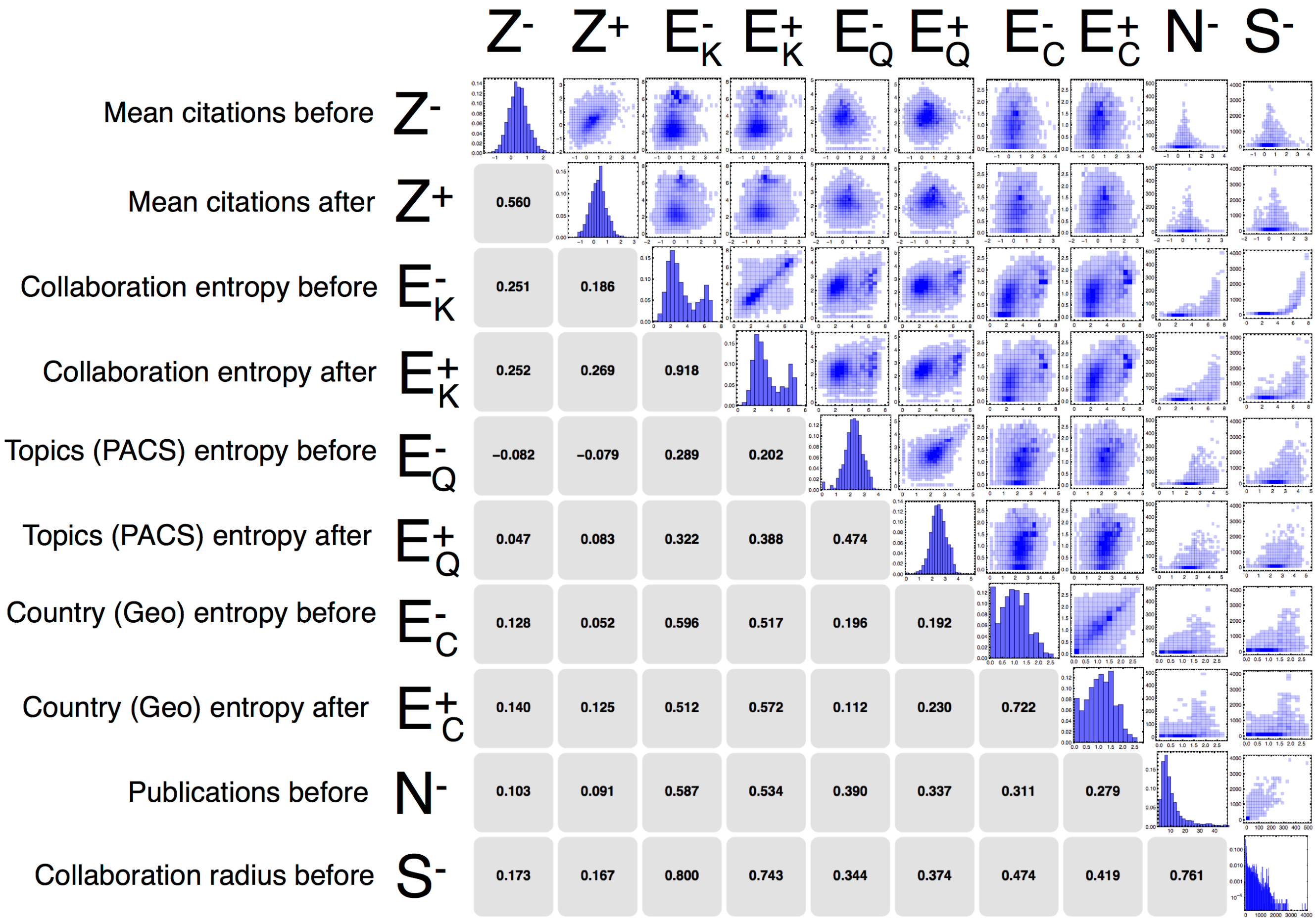

FIG. S7: **Model variables – distribution and covariation.** Shown is the correlation matrix calculated using the variables included in the PSM model; data are combined across the three periods (T). The diagonal elements show the distribution of the variable quantities; the upper-diagonal elements show the density-weighted scatter plots of any given pair of data observations; the lower-diagonal elements list the Pearson correlation coefficient between the corresponding variable pairs.

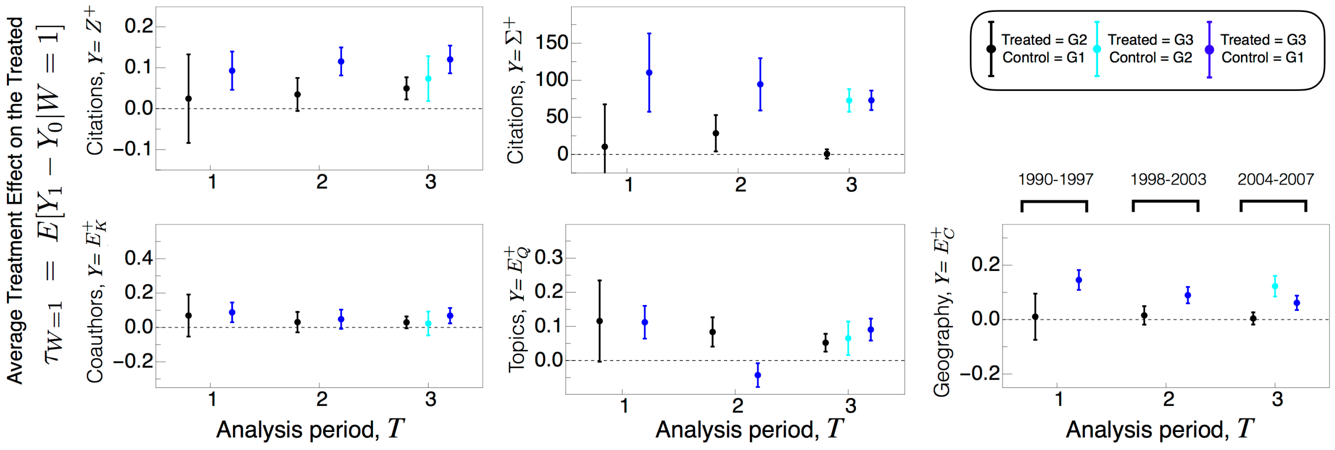

FIG. S8: **Estimation of the mobility effect using the nearest-neighbor *nnmatch* matching method**. Robustness check for the propensity score matching results reported in Fig. 3. The *teffects nnmatch* routine differs from the *teffects psmatch* in that the former calculates a single distance between multi-variate observations using a the Mahalanobis metric, and then matches to the nn closest observations (we used nn=1) [52]. One particular advantage of the *teffects nnmatch* method is that it allows the option to force a match on specified variables (using the ematch option); hence, we forced matches on the geographic region factor variable F_i^- representing one of the 5 geographic (continental) regions that the researcher primarily resided in prior to $t^*_{i,T}$ (see "Country classification" in Section S3). In this capacity, the *teffects nnmatch* estimate appropriately matches mobile individuals to un-mobile individuals from the same geographic region, thereby controlling for variation in regional migration opportunity. Despite this key difference, each set of estimates are robust with respect to the *teffects psmatch* estimates with the exception of the coauthor analysis (bottom row, left panel). Each error bar is a point estimate with 95% confidence interval.

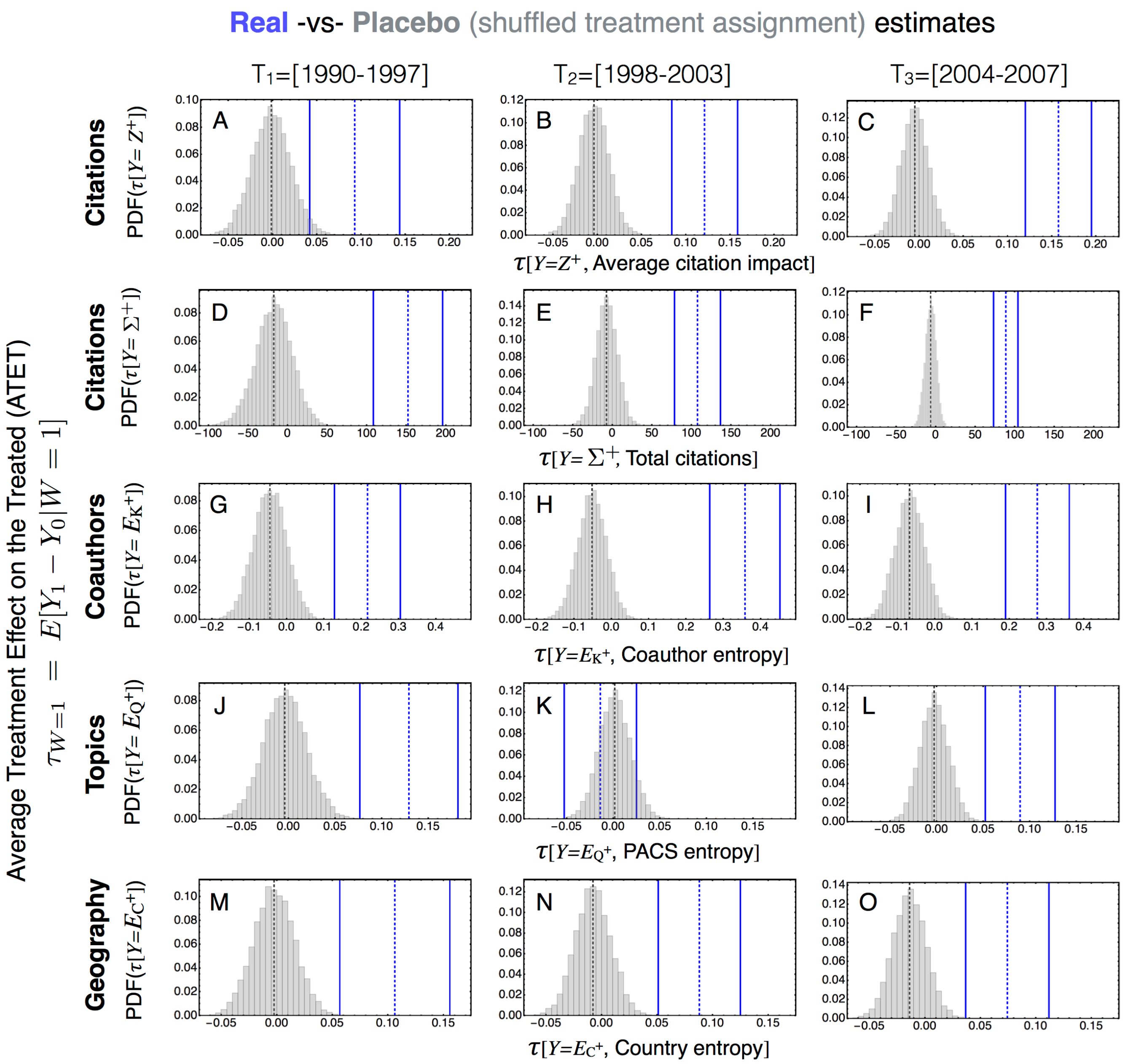

FIG. S9: **Testing the statistical significance of** $\tau_{W=1}$. It is possibility that spurious correlations could give rise to the statistically significant PSM estimations for $\tau_{W=1}[Y]$ reported in Figs. 3 and S8. We explored this possibility for the PSM models comparing $G1$ (control) and $G3$ (mobility) groups by randomizing the group assignments, implemented by shuffling G_i without replacement so that the total number of researchers in each group is conserved relative to the unshuffled (real) data. Thus, for each dependent variable (Y), we produced $N = 10,000$ shuffled datasets ('placebo model'), calculating $\tau_{W=1}[Y]$ for each. Shown for each specification is the probability distribution $P(\tau_{W=1})$ of the placebo estimates for $\tau_{W=1}[Y]$; the solid vertical blue line indicates the real $\tau_{W=1}[Y]$, and the dashed lines indicate the corresponding 95% confidence interval. In all cases except for in panel K, in which $\tau_{W=1}[E_{Q,T2}]$ is not statistically significant in the first place, we can rule out the possibility that $\tau_{W=1}[Y]$ estimations are statistically significant due to chance.

TABLE S1: Logit model. The dependent variable of the model is the binary outcome variable $1_{G_i=3}$ with value 1 if researcher i migrated during T and value 0 if there was no migration during or before T. Reported are odds ratios, $\exp(\beta)$.

	$T_1 = [1990-1997]$		$T_2 = [1998-2003]$		$T_3 = [2004-2007]$	
**Researcher variables**						
coauthors, $\lvert k_{ij}^{-}\rvert$	0.996***	(0.000)	0.999***	(0.000)	0.999***	(0.000)
publications, N_i^{-}	1.023***	(0.000)	1.005*	(0.037)	1.001	(0.247)
citation impact Z_i^{-}	1.132*	(0.043)	1.155***	(0.001)	0.969	(0.449)
researcher age, s_i^*	0.781***	(0.000)	0.856***	(0.000)	0.899***	(0.000)
**Researcher geographic region,** F_i^{-}						
N. America	1	(.)	1	(.)	1	(.)
S. & C. America	0.871	(0.591)	0.400***	(0.000)	0.383***	(0.000)
Europe	0.455***	(0.000)	0.492***	(0.000)	0.452***	(0.000)
Asia & Australasia	0.373***	(0.000)	0.350***	(0.000)	0.361***	(0.000)
N	4117		9347		13446	

p-values in parentheses.

* $p < 0.05$, ** $p < 0.01$, *** $p < 0.001$

TABLE S2: **Results of OLS regression using matched researcher pairs** (i, i'). The dependent variable is $Y_i^{+} \equiv Z_i^{+}$, the average citation impact after $t_{i,T}^*$.

	(1)		(2)		(3)		(4)		(5)		(6)	
	T_1 = [1990–1997]		T_1 = [1990–1997]		T_2 = [1990–1997]		T_2 = [1998–2003]		T_3 = [2004–2007]		T_3 = [2004–2007]	
			(w/ $1_{G_i=3}$ interaction)				(w/ $1_{G_i=3}$ interaction)				(w/ $1_{G_i=3}$ interaction)	
coauthors, $\lvert k_{ij}^{-}\rvert$	0.000***	(0.000)	0.000	(0.099)	0.000***	(0.000)	0.000***	(0.000)	0.000***	(0.000)	0.000***	(0.000)
# interaction difference, $\delta_3(\lvert k_{ij}^{-}\rvert)$			0.000	(0.052)			-0.000*	(0.047)			-0.000	(0.055)
publications, N_i^{-}	-0.000	(0.885)	0.004*	(0.022)	-0.003***	(0.000)	-0.004***	(0.000)	-0.001	(0.137)	-0.000	(0.515)
# interaction difference, $\delta_3(N_i^{-})$			-0.009***	(0.000)			0.002	(0.105)			-0.000	(0.848)
citation impact, Z_i^{-}	0.486***	(0.000)	0.585***	(0.000)	0.460***	(0.000)	0.540***	(0.000)	0.465***	(0.000)	0.495***	(0.000)
# interaction difference, $\delta_3(Z_i^{-})$			-0.195***	(0.000)			-0.160***	(0.000)			-0.069**	(0.004)
researcher age, s_i^*	-0.009	(0.062)	-0.005	(0.393)	-0.006**	(0.003)	0.003	(0.370)	-0.011***	(0.000)	-0.007***	(0.001)
# interaction difference, $\delta_3(s_i^*)$			-0.008	(0.379)			-0.017***	(0.000)			-0.007*	(0.032)
Mobile researcher indicator $(1_{G_i=3})$	0.088***	(0.000)	0.286***	(0.000)	0.124***	(0.000)	0.325***	(0.000)	0.114***	(0.000)	0.231***	(0.000)
Constant	0.098***	(0.001)	0.004	(0.919)	0.162***	(0.000)	0.056*	(0.039)	0.170***	(0.000)	0.115***	(0.000)
Researcher geo. region fixed effect, F_i^{-}	Y		Y		Y		Y		Y		Y	
N	3342		3342		5048		5048		4600		4600	
adj. R^2	0.274		0.289		0.267		0.275		0.306		0.309	
F	158.903		114.212		230.241		160.577		254.016		172.004	

p-values in parentheses

* $p < 0.05$, ** $p < 0.01$, *** $p < 0.001$

TABLE S3: **Results of OLS regression using matched researcher pairs** (i, i'). The dependent variable is $Y_i^+ \equiv \Sigma_i^+$, the total deflated citations after $t^*_{i,T}$.

	(1)		(2)		(3)		(4)		(5)		(6)	
	T_1 = [1990–1997]		T_1 = [1990–1997] (w/ $1_{G_i=3}$ interaction)		T_2 = [1990–1997]		T_2 = [1998–2003] (w/ $1_{G_i=3}$ interaction)		T_3 = [2004–2007]		T_3 = [2004–2007] (w/ $1_{G_i=3}$ interaction)	
total deflated citations, Σ_i^-	0.353***	(0.000)	0.446***	(0.000)	0.099***	(0.001)	0.311***	(0.000)	0.175***	(0.000)	-0.007	(0.808)
# interaction difference, $\delta_3(\Sigma_i^-)$			-0.195*	(0.032)			-0.395***	(0.000)			0.307***	(0.000)
coauthors, $\|k_{ij}^-\|$	3.849***	(0.000)	3.995***	(0.000)	1.500***	(0.000)	1.185***	(0.000)	0.389***	(0.000)	0.264***	(0.000)
# interaction difference, $\delta_3(\|k_{ij}^-\|)$			-0.219	(0.259)			0.433***	(0.000)			0.192***	(0.000)
publications, N_i^-	-8.839***	(0.000)	-13.611***	(0.000)	-1.844*	(0.011)	-5.654***	(0.000)	0.413	(0.185)	3.794***	(0.000)
# interaction difference, $\delta_3(N_i^-)$			8.714*	(0.025)			7.853***	(0.000)			-5.508***	(0.000)
citation impact, Z_i^-	23.215	(0.181)	22.389	(0.362)	34.774**	(0.004)	8.535	(0.621)	-16.293*	(0.017)	25.608**	(0.009)
# interaction difference, $\delta_3(Z_i^-)$			7.748	(0.822)			49.320*	(0.036)			-68.474***	(0.000)
researcher age, s_i^*	-29.642***	(0.000)	-22.662***	(0.000)	0.679	(0.691)	1.005	(0.672)	-4.492***	(0.000)	-3.841***	(0.000)
# interaction difference, $\delta_3(s_i^*)$			-14.780	(0.082)			-2.855	(0.402)			-1.160	(0.402)
Mobile researcher indicator $(1_{G_i=3})$	107.441***	(0.000)	165.309***	(0.001)	68.059***	(0.000)	30.758	(0.298)	60.188***	(0.000)	84.689***	(0.000)
Constant	253.634***	(0.000)	229.265***	(0.000)	115.496***	(0.000)	147.993***	(0.000)	32.515***	(0.001)	15.183	(0.197)
Researcher geo. region fixed effect, F_i^-	Y		Y		Y		Y		Y		Y	
N	3342		3342		5048		5048		4600		4600	
adj. R^2	0.527		0.528		0.358		0.369		0.489		0.502	
F	415.097		267.964		313.401		212.108		489.345		331.721	

p-values in parentheses
* $p < 0.05$, ** $p < 0.01$, *** $p < 0.001$

TABLE S4: **Results of OLS regression using matched researcher pairs** (i, i'). The dependent variable is $Y_i^+ \equiv E_{K,i}^+$, the coauthor entropy after $t^*_{i,T}$.

	(1)		(2)		(3)		(4)		(5)		(6)	
	T_1 = [1990–1997]		T_1 = [1990–1997] (w/ $1_{G_i=3}$ interaction)		T_2 = [1990–1997]		T_2 = [1998–2003] (w/ $1_{G_i=3}$ interaction)		T_3 = [2004–2007]		T_3 = [2004–2007] (w/ $1_{G_i=3}$ interaction)	
coauthor entropy, $E_{K,i}^-$	0.848***	(0.000)	0.804***	(0.000)	0.865***	(0.000)	0.766***	(0.000)	0.844***	(0.000)	0.840***	(0.000)
# interaction difference, $\delta_3(E_{K,i}^-)$			0.087**	(0.003)			0.155***	(0.000)			0.009	(0.652)
coauthors, $\|k_{ij}^-\|$	0.001***	(0.000)	0.002***	(0.000)	0.001***	(0.000)	0.002***	(0.000)	0.000***	(0.000)	0.000***	(0.000)
# interaction difference, $\delta_3(\|k_{ij}^-\|)$			-0.001**	(0.008)			-0.002***	(0.000)			-0.000	(0.229)
publications, N_i^-	-0.008***	(0.000)	-0.009***	(0.001)	-0.003***	(0.001)	-0.008***	(0.000)	-0.001	(0.182)	-0.001	(0.090)
# interaction difference, $\delta_3(N_i^-)$			0.003	(0.378)			0.006**	(0.006)			0.001	(0.313)
citation impact, Z_i^-	-0.049*	(0.015)	-0.019	(0.509)	-0.114***	(0.000)	-0.110***	(0.000)	0.047**	(0.005)	0.023	(0.342)
# interaction difference, $\delta_3(Z_i^-)$			-0.056	(0.162)			0.008	(0.815)			0.048	(0.152)
researcher age, s_i^*	-0.036***	(0.000)	-0.030***	(0.000)	-0.027***	(0.000)	-0.027***	(0.000)	-0.022***	(0.000)	-0.022***	(0.000)
# interaction difference, $\delta_3(s_i^*)$			-0.016	(0.198)			0.006	(0.344)			-0.001	(0.736)
Mobile researcher indicator $(1_{G_i=3})$	0.084***	(0.000)	0.010	(0.907)	0.036	(0.102)	-0.355***	(0.000)	0.075***	(0.000)	0.040	(0.517)
Constant	0.966***	(0.000)	1.010***	(0.000)	1.039***	(0.000)	1.256***	(0.000)	0.807***	(0.000)	0.825***	(0.000)
Researcher geo. region fixed effect, F_i^-	Y		Y		Y		Y		Y		Y	
N	3342		3342		5048		5048		4600		4600	
adj. R^2	0.776		0.776		0.767		0.769		0.852		0.852	
F	1285.405		828.658		1847.981		1202.122		2946.382		1894.009	

p-values in parentheses
* $p < 0.05$, ** $p < 0.01$, *** $p < 0.001$

TABLE S5: **Results of OLS regression using matched researcher pairs** (i, i'). The dependent variable is $Y_i^+ \equiv E_{Q,i}^+$, the PACS (research topic) entropy after $t_{i,T}^*$.

	(1)	(2)	(3)	(4)	(5)	(6)
	T_1 = [1990–1997]	T_1 = [1990–1997] (w/ $1_{G_i=3}$ interaction)	T_2 = [1990–1997]	T_2 = [1998–2003] (w/ $1_{G_i=3}$ interaction)	T_3 = [2004–2007]	T_3 = [2004–2007] (w/ $1_{G_i=3}$ interaction)
PACS (research topic) entropy, $E_{Q,i}^-$	0.336*** (0.000)	0.397*** (0.000)	0.243*** (0.000)	0.312*** (0.000)	0.458*** (0.000)	0.481*** (0.000)
# interaction difference, $\delta_3(E_{Q,i}^-)$		-0.114** (0.004)		-0.131*** (0.000)		-0.048 (0.106)
coauthors, $\|k_{ij}^-\|$	0.001*** (0.000)	0.002*** (0.000)	0.001*** (0.000)	0.001*** (0.000)	0.000*** (0.000)	0.000*** (0.000)
# interaction difference, $\delta_3(\|k_{ij}^-\|)$		-0.001** (0.004)		-0.000*** (0.001)		0.000 (0.116)
publications, N_i^-	0.002 (0.136)	0.000 (0.970)	-0.001 (0.089)	-0.005*** (0.000)	0.000 (0.906)	0.000 (0.989)
# interaction difference, $\delta_3(N_i^-)$		0.004 (0.164)		0.006*** (0.000)		0.000 (0.899)
citation impact, Z_i^-	-0.038* (0.021)	-0.059* (0.011)	0.096*** (0.000)	0.170*** (0.000)	0.010 (0.400)	0.006 (0.740)
# interaction difference, $\delta_3(Z_i^-)$		0.047 (0.145)		-0.139*** (0.000)		0.011 (0.643)
researcher age, s_i^*	-0.039*** (0.000)	-0.025*** (0.000)	-0.002 (0.487)	-0.003 (0.384)	-0.006*** (0.000)	-0.009*** (0.000)
# interaction difference, $\delta_3(s_i^*)$		-0.032** (0.002)		0.005 (0.235)		0.006 (0.069)
Mobile researcher indicator $(1_{G_i=3})$	0.123*** (0.000)	0.501*** (0.000)	-0.024 (0.111)	0.246*** (0.000)	0.083*** (0.000)	0.121 (0.078)
Constant	1.629*** (0.000)	1.435*** (0.000)	1.847*** (0.000)	1.689*** (0.000)	1.363*** (0.000)	1.343*** (0.000)
Researcher geo. region fixed effect, F_i^-	Y	Y	Y	Y	Y	Y
N	3342	3342	5048	5048	4600	4600
adj. R^2	0.204	0.212	0.148	0.157	0.330	0.331
F	96.397	65.195	98.686	67.912	252.875	163.279

p-values in parentheses
$^* p < 0.05$, $^{**} p < 0.01$, $^{***} p < 0.001$

TABLE S6: **Results of OLS regression using matched researcher pairs** (i, i'). The dependent variable is $Y_i^+ \equiv E_{C,i}^+$, the country entropy after $t_{i,T}^*$.

	(1)	(2)	(3)	(4)	(5)	(6)
	T_1 = [1990–1997]	T_1 = [1990–1997] (w/ $1_{G_i=3}$ interaction)	T_2 = [1990–1997]	T_2 = [1998–2003] (w/ $1_{G_i=3}$ interaction)	T_3 = [2004–2007]	T_3 = [2004–2007] (w/ $1_{G_i=3}$ interaction)
country entropy, $E_{C,i}^-$	0.466*** (0.000)	0.553*** (0.000)	0.536*** (0.000)	0.632*** (0.000)	0.597*** (0.000)	0.721*** (0.000)
# interaction difference, $\delta_3(E_{C,i}^-)$		-0.182*** (0.000)		-0.227*** (0.000)		-0.265*** (0.000)
coauthors, $\|k_{ij}^-\|$	0.001*** (0.000)	0.001*** (0.000)	0.001*** (0.000)	0.001*** (0.000)	0.000*** (0.000)	0.000* (0.017)
# interaction difference, $\delta_3(\|k_{ij}^-\|)$		0.000 (0.107)		0.000 (0.969)		0.000*** (0.000)
publications, N_i^-	-0.003** (0.005)	-0.001 (0.508)	0.001 (0.249)	0.000 (0.604)	0.000 (0.241)	0.000 (0.381)
# interaction difference, $\delta_3(N_i^-)$		-0.004 (0.080)		-0.000 (0.897)		-0.000 (0.673)
citation impact, Z_i^-	0.013 (0.293)	0.042* (0.023)	-0.015 (0.147)	-0.008 (0.573)	0.086*** (0.000)	0.129*** (0.000)
# interaction difference, $\delta_3(Z_i^-)$		-0.059* (0.019)		-0.011 (0.566)		-0.092*** (0.000)
researcher age, s_i^*	-0.005 (0.164)	-0.007 (0.168)	-0.006*** (0.001)	-0.008** (0.002)	-0.008*** (0.000)	-0.009*** (0.000)
# interaction difference, $\delta_3(s_i^*)$		0.005 (0.544)		0.007 (0.059)		0.004 (0.167)
Mobile researcher indicator $(1_{G_i=3})$	0.114*** (0.000)	0.237*** (0.000)	0.084*** (0.000)	0.244*** (0.000)	0.035** (0.002)	0.293*** (0.000)
Constant	0.447*** (0.000)	0.382*** (0.000)	0.460*** (0.000)	0.372*** (0.000)	0.352*** (0.000)	0.229*** (0.000)
Researcher geo. region fixed effect, F_i^-	Y	Y	Y	Y	Y	Y
N	3342	3342	5048	5048	4600	4600
adj. R^2	0.328	0.335	0.426	0.438	0.536	0.552
F	182.449	121.347	417.520	282.008	590.838	406.136

p-values in parentheses
$^* p < 0.05$, $^{**} p < 0.01$, $^{***} p < 0.001$
